# Cultivation and sequencing of rumen microbiome members from the Hungate1000 Collection

**DOI:** 10.1038/nbt.4110

**Published:** 2018-03-19

**Authors:** Rekha Seshadri, Sinead C Leahy, Graeme T Attwood, Koon Hoong Teh, Suzanne C Lambie, Adrian L Cookson, Emiley A Eloe-Fadrosh, Georgios A Pavlopoulos, Michalis Hadjithomas, Neha J Varghese, David Paez-Espino, Hungate1000 project collaborators, Nikola Palevich, Peter H Janssen, Ron S Ronimus, Samantha Noel, Priya Soni, Kerri Reilly, Todd Atherly, Cherie Ziemer, Andre-Denis Wright, Suzanne Ishaq, Michael Cotta, Stephanie Thompson, Katie Crosley, Nest McKain, R John Wallace, Harry J Flint, Jennifer C Martin, Robert J Forster, Robert J Gruninger, Tim McAllister, Rosalind Gilbert, Diane Ouwerkerk, Athol Klieve, Rafat Al Jassim, Stuart Denman, Chris McSweeney, Carly Rosewarne, Satoshi Koike, Yasuo Kobayashi, Makoto Mitsumori, Takumi Shinkai, Silvio Cravero, María Cerón Cucchi, Rechelle Perry, Gemma Henderson, Christopher J Creevey, Nicolas Terrapon, Pascal Lapebie, Elodie Drula, Vincent Lombard, Edward Rubin, Nikos C Kyrpides, Bernard Henrissat, Tanja Woyke, Natalia N Ivanova, William J Kelly

**Affiliations:** 1 Department of Energy, Joint Genome Institute, Walnut Creek, California USA; 2 AgResearch Limited, Grasslands Research Centre, Palmerston North, New Zealand; 4 Institute of Biological, Environmental and Rural Sciences, Aberystwyth University, Aberystwyth, Wales UK; 5 Architecture et Fonction des Macromolécules Biologiques, Aix-Marseille Université, Marseille, France; 6 Institut National de la Recherche Agronomique, Marseille, France; 7 Department of Biological Sciences, King Abdulaziz University, Jeddah, Saudi Arabia; 10 AgResearch Limited, Grasslands Research Centre, Palmerston North, New Zealand; 11 ARS-USDA National Laboratory for Agriculture and the Environment, Ames, Iowa USA; 12 School of Animal and Comparative Biomedical Sciences, the University of Arizona, Tucson, Arizona USA; 13 Biology and the Built Environment Center, University of Oregon, Eugene, Oregon USA; 14 ARS-USDA National Center for Agricultural Utilization Research, Peoria, Illinois USA; 15 Rowett Institute, University of Aberdeen, Aberdeen, Scotland, UK., ,; 16 Agriculture & Agri-Food Canada, Lethbridge Research & Development Centre, Lethbridge, Alberta Canada; 17 Department of Agriculture and Fisheries, Agri-Science Queensland, Brisbane, Australia; 18 Queensland Alliance for Agriculture and Food Innovation, University of Queensland, Brisbane, Australia; 19 School of Agriculture and Food Sciences, University of Queensland, Brisbane, Australia; 20 CSIRO Agriculture and Food, St. Lucia, Queensland Australia; 21 CSIRO Health and Biosecurity, Adelaide, South Australia Australia; 22 Research Faculty of Agriculture, Hokkaido University, Sapporo, Japan; 23 National Institute of Livestock and Grassland Science, Tsukuba, Japan; 24 Instituto Nacional de Tecnología Agropecuaria, Hurlingham, Argentina; 25 Present Address: New Zealand Agricultural Greenhouse Gas Research Centre, Palmerston North, New Zealand; 26 Present Address: Massey University, Auckland, New Zealand; 27 Present Address: Chr. Hansen A/S, Hørsholm, Denmark; 28 Present Address: Metabiota, San Francisco California USA; 29 Present Address: Donvis Ltd, Palmerston North, New Zealand; 30 Present Address: Hill Laboratories, Blenheim, New Zealand

**Keywords:** Genome, Microbiology, Ecology, Computational biology and bioinformatics, Microbial ecology

## Abstract

Productivity of ruminant livestock depends on the rumen microbiota, which ferment indigestible plant polysaccharides into nutrients used for growth. Understanding the functions carried out by the rumen microbiota is important for reducing greenhouse gas production by ruminants and for developing biofuels from lignocellulose. We present 410 cultured bacteria and archaea, together with their reference genomes, representing every cultivated rumen-associated archaeal and bacterial family. We evaluate polysaccharide degradation, short-chain fatty acid production and methanogenesis pathways, and assign specific taxa to functions. A total of 336 organisms were present in available rumen metagenomic data sets, and 134 were present in human gut microbiome data sets. Comparison with the human microbiome revealed rumen-specific enrichment for genes encoding *de novo* synthesis of vitamin B_12_, ongoing evolution by gene loss and potential vertical inheritance of the rumen microbiome based on underrepresentation of markers of environmental stress. We estimate that our Hungate genome resource represents ∼75% of the genus-level bacterial and archaeal taxa present in the rumen.

**Supplementary information:**

The online version of this article (doi:10.1038/nbt.4110) contains supplementary material, which is available to authorized users.

## Abstract (web summary)

Rumen microbiome biology gets a boost with the release of 410 high-quality reference genomes from the Hungate1000 project.

**Supplementary information:**

The online version of this article (doi:10.1038/nbt.4110) contains supplementary material, which is available to authorized users.

## Main

Climate change and feeding a growing global population are the two biggest challenges facing agriculture^1^. Ruminant livestock have an important role in food security^2^; they convert low-value lignocellulosic plant material into high-value animal proteins that include milk, meat and fiber products. Microorganisms present in the rumen^3,4^ ferment polysaccharides to yield short-chain fatty acids (SCFAs; acetate, butyrate and propionate) that are absorbed across the rumen epithelium and used by the ruminant for maintenance and growth. The rumen represents one of the most rapid and efficient lignocellulose depolymerization and utilization systems known, and is a promising source of enzymes for application in lignocellulose-based biofuel production^5^. Enteric fermentation in ruminants is also the single largest anthropogenic source of methane (CH_4_)^6^, and each year these animals release ∼125 million tonnes of CH_4_ into the atmosphere. Targets to reduce agricultural carbon emissions have been proposed^7^, with >100 countries pledging to reduce agricultural greenhouse gas emissions in the 2015 Paris Agreement of the United Nations Framework Convention on Climate Change. Consequently, improved knowledge of the flow of carbon through the rumen by lignocellulose degradation and fermentation to SCFAs and CH_4_ is relevant to food security, sustainability and greenhouse gas emissions.

Understanding the functions of the rumen microbiome is crucial to the development of technologies and practices that support efficient global food production from ruminants while minimizing greenhouse gas emissions. The Rumen Microbial Genomics Network (http://www.rmgnetwork.org/) was launched under the auspices of the Livestock Research Group of the Global Research Alliance (http://globalresearchalliance.org/research/livestock/) to further this understanding, with the generation of a reference microbial genome catalog—the Hungate1000 project—as a primary collaborative objective. Although the microbial ecology of the rumen has long been the focus of research^8,9^, at the beginning of the project reference genomes were available for only 14 bacteria and one methanogen, so that genomic diversity was largely unexplored.

The Hungate1000 project was initiated as a community resource in 2012, and the collection assembled includes virtually all the bacterial and archaeal species that have been cultivated from the rumens of a diverse group of animals^10^. We surveyed Members of the Rumen Microbial Genomics Network and requested they provide cultures of interest. We supplemented these with additional cultures purchased from culture collections to generate the most comprehensive collection possible. These cultures are available to researchers, and we envisage that additional organisms will have their genome sequences included as more rumen microbes are able to be cultivated.

Large-scale reference genome catalogs, including the Human Microbiome Project (HMP)^11^ and the Genomic Encyclopedia of Bacteria and Archaea (GEBA)^12^ have helped to improve our understanding of microbiome functions, diversity and interactions with the host. The success of these efforts has resulted in calls for continued development of high-quality reference genome catalogs^13,14^, and led to a resurgence in efforts to cultivate microorganisms^15,16,17^. This high-quality reference genome catalog for rumen bacteria and archaea increases our understanding of rumen functions by revealing degradative and physiological capabilities, and identifying potential rumen-specific adaptations.

## Results

### Reference rumen genomes

Members of nine phyla, 48 families and 82 genera (Supplementary Table 1 and Supplementary Note 1) are present in the Hungate Collection. The organisms were chosen to make the coverage of cultivated rumen microbes as comprehensive as possible^10^. While multiple isolates were sequenced from some polysaccharide-degrading genera (*Butyrivibrio*, *Prevotella* and *Ruminococcus*), many species are represented by only one or a few isolates. 410 reference genomes were sequenced in this study, and were analyzed in combination with 91 publicly available genomes^18^. All Hungate1000 genomes were sequenced using Illumina or PacBio technology, and were assembled and annotated as summarized in the Online Methods. All genomes were assessed as high quality using CheckM^19^ with >99% completeness on average, and in accordance with proposed standards^20^. The genome statistics can be found in Supplementary Table 2.

The 501 sequenced organisms analyzed in this study are listed in Supplementary Table 1. We refer to these 501 genomes (480 bacteria and 21 archaea) as the Hungate genome catalog. Supplementary Table 3 provides a comprehensive chronological list of all publicly available completed rumen microbial genome sequencing projects, including anaerobic fungi and genomes that have been recovered from metagenomes but that were not included in our analyses.

Members of the Firmicutes and Bacteroidetes phyla predominate in the rumen^21,22^ and contribute most of the Hungate genome sequences (68% and 12.8%, respectively; Supplementary Fig. 1a), with the *Lachnospiraceae* family making up the largest single group (32.3%). Archaea are mainly from the *Methanobrevibacter* genus or are in the *Methanomassiliicoccales* order. The average genome size is ∼3.3 Mb (Supplementary Fig. 1b), and the average G+C content is 44%. Most organisms were isolated directly from the rumen (86.6%), with the remainder isolated from feces or saliva. Most cultured organisms were from bovine (70.9%) or ovine (17.6%) hosts, but other ruminant or camelid species are also represented (Table 1).

**Table 1:** Hungate1000 Collection

Phylum	No. of cultures	Livestock source	No. of cultures	Country of origin	No. of cultures
Actinobacteria	33	Bison	1	Argentina	4
Bacteroidetes	64	Buffalo	3	Australia	44
Euryarchaeota	21	Calf	20	Canada	3
Fibrobacteres	2	Camel	8	China	5
Firmicutes	341	Cow	337	Czech republic	1
Fusobacteria	1	Deer	4	France	1
Proteobacteria	31	Goat	21	Germany	3
Spirochaetes	6	Goose	1	India	4
Synergistetes	2	Horse	2	Ireland	1
		Lamb	4	Italy	7
		Llama	4	Japan	19
		Moose	8	Korea	5
		Pig	1	Malaysia	1
		Sheep	84	New Zealand	258
		Yak	3	Slovenia	1
				South Africa	6
				Spain	1
				Sweden	9
				Switzerland	1
				UK	27
				USA	100

Table 1 is expanded in Supplementary Table 1 and Supplementary Note 1.

**Figure 1:**
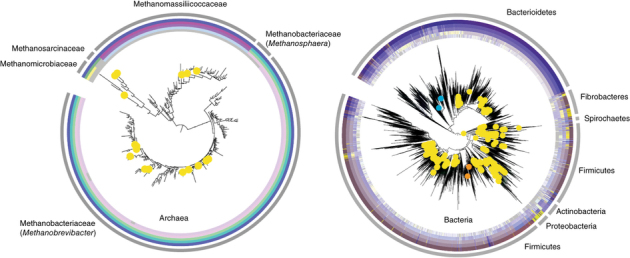
Microbial community composition data from the Global Rumen Census^22^ overlaid with the 16S rRNA gene sequences (yellow dots) from the 501 Hungate catalog genomes. Two groups of abundant but currently unclassified bacteria are indicated by blue (*Bacteroidales*, RC-9 gut group) and orange (*Clostridiales*, R-7 group) dots. The colored rings around the trees represent the taxonomic classifications of each OTU from the Ribosomal Database Project database (from the innermost to the outermost): genus, family, order, class and phylum. The strength of the color is indicative of the percentage similarity of the OTU to a sequence in the RDP database of that taxonomic level.

The Global Rumen Census project^22^ profiled the microbial communities of 742 rumen samples present in diverse ruminant species, and found that rumen communities largely comprised similar bacteria and archaea in the 684 samples that met the criteria for inclusion in the analysis. A core microbiome of seven abundant genus-level groups was defined for 67% of the Global Rumen Census sequences^22^. We overlaid 16S rRNA gene sequences from the 501 Hungate genomes onto the 16S rRNA gene amplicon data set from the Global Rumen Census project (Fig. 1). This revealed that our Hungate genomes represent ∼75% of the genus-level taxa reported from the rumen.

Previous studies of the rumen microbiome have highlighted unclassified bacteria as being among the most abundant rumen microorganisms^10,21^, and we also report 73 genome sequences from strains that have yet to be taxonomically assigned to genera or phenotypically characterized (Supplementary Table 1). Most abundant among these uncharacterized strains are members of the order *Bacteroidales* (RC-9 gut group) and *Clostridiales* (R-7 group), and this abundance points to a key role for these strains in rumen fermentation^22^. The RC-9 gut group bacteria have small genomes (∼2.3 Mb), and the closest named relatives (84% identity of the 16S rRNA gene) are members of the genus *Alistipes*, family *Rikenellaceae*. The R-7 group are most closely related to *Christensenella minuta* (86% identity of the 16S rRNA gene), family *Christensenellaceae*.

### Functions of the rumen microbiome

#### Polysaccharide degradation.

Ruminants need efficient lignocellulose breakdown to satisfy their energy requirements, but ruminant genomes, in common with the human genome, encode very limited degradative enzyme capacity. Cattle have a single pancreatic amylase^23^, and several lysozymes^24^ which functions as lytic digestive enzymes that can kill Gram-positive bacteria^25^.

**Figure 2:**
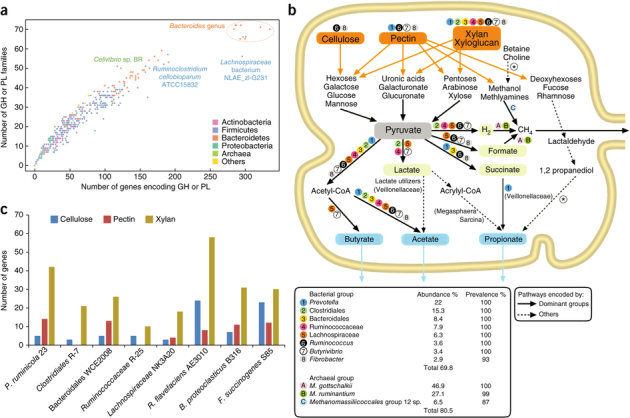
Functions of the rumen microbiome. (**a**) Number of degradative CAZymes (GH, glycoside hydrolases and PL, polysaccharide lyases) in distinct families in each of the 501 Hungate catalog genomes. Genomes are colored by phylum. (**b**) Simplified illustration showing the degradation and metabolism of plant structural carbohydrates by the dominant bacterial and archaeal groups identified in the Global Rumen Census project^22^ using information from metabolic studies and analysis of the reference genomes. The abundance and prevalence data shown in the table are taken from the Global Rumen Census project^22^. Abundance represents the mean relative abundance (%) for that genus-level group in samples that contain that group, while prevalence represents the prevalence of that genus-level group in all samples (*n* = 684).* The conversion of choline to trimethylamine, and propanediol to propionate generate toxic intermediates that are contained within bacterial microcompartments (BMC). Cultures from the reference genome set that encode the genes required to produce the structural proteins required for BMC formation are shown in Supplementary Table 5. (**c**) Number of polysaccharide-degrading CAZymes encoded in the genomes of representatives from the eight most abundant bacterial groups. Cellulose: GH5, GH9, GH44, GH45, GH48; pectin: GH28, PL1, PL9, PL10, PL11, CE8, CE12; xylan: GH8, GH10, GH11, GH43, GH51, GH67, GH115, GH120, GH127, CE1, CE2.

We searched the CAZy database for each Hungate genome (http://www.cazy.org/)^26^ in order to characterize the spectrum of carbohydrate-active enzymes and binding proteins present (Supplementary Fig. 2 and Supplementary Table 4). In total, the Hungate genomes encode 32,755 degradative CAZymes (31,569 glycoside hydrolases and 1,186 polysaccharide lyases), representing 2.2% of the combined ORFeome. The largest and most diverse CAZyme repertoires (Fig. 2a) were found in isolates with large genomes including *Bacteroides ovatus* (over 320 glycoside hydrolases (GH) and polysaccharide lyases (PL) from ∼60 distinct families), *Lachnospiraceae* bacterium NLAE-zl-G231 (296 GHs and PLs), *Ruminoclostridium cellobioparum* ATCC 15832 (184 GHs and PLs) and *Cellvibrio* sp. BR (158 GHs and PLs). The most prevalent CAZyme families are shown in Supplementary Figure 3. Bacteria that initiate the breakdown of plant fiber are predicted to be important in rumen microbial fermentation (Fig. 2b), including representatives of bacterial groups capable of degrading cellulose, hemicellulose (xylan/xyloglucan) and pectin (Fig. 2c).

Examination of the CAZyme profiles (Supplementary Fig. 3) highlights the degradation strategies used by different taxa present in our collection. Members of the phylum Bacteroidetes have evolved polysaccharide utilization loci (PULs), genomic regions that encode all required components for the binding, transport and depolymerization of specific glycan structures. Predictions of PUL organization in all 64 Bacteroidetes genomes from the Hungate catalog have been integrated into the dedicated PULDB database^27^. The pectin component rhamnogalacturonan II (RG-II) is the most structurally complex plant polysaccharide, and all the CAZymes required for its degradation occur in a single large PUL recently identified in *Bacteroides thetaiotaomicron*^28^. Similar PULs encoding all necessary enzymes were also found in rumen isolates belonging to three different families within the phylum Bacteroidetes (Supplementary Fig. 2 and Supplementary Fig. 4). Another feature of the Bacteroidetes genomes and PULs is the prevalence of GH families dedicated to the breakdown of animal glycans (Supplementary Figure 2). Host glycans are not thought to be used as a carbohydrate source for rumen bacteria, and most of the genomes with extensive repertoires of these enzymes (*Bacteroides* spp.) were from species that were isolated from feces. However, ruminants secrete copious saliva and the presence of animal glycan-degrading enzymes in rumen *Prevotella* spp. may enable them to utilize salivary N-linked glycoproteins^29^, and help explain their abundance in the rumen microbiome^22^.

The multisubunit cellulosome is an alternative strategy for complex glycan breakdown in which a small module (dockerin) appended to glycan-cleaving enzymes anchors various catalytic units onto cognate cohesin repeats found on a large scaffolding protein^30^. Cellulosomes have been reported in only a small number of species, mainly in the family *Ruminococcaceae* in the order *Clostridiales*. Supplementary Table 4 reports the number of dockerin and cohesin modules found in the reference genomes and the main cellulosomal bacteria are highlighted in Supplementary Figure 2. We find that *Clostridiales* bacteria can be divided into four broad categories: (i) those that have neither dockerins nor cohesins (non-cellulosomal species), (ii) those that have just a few dockerins and no cohesins (most likely non-cellulosomal), (iii) those that have a large number of dockerins and many cohesins (true cellulosomal bacteria like *Ruminococcus flavefaciens*) and (iv) those that have a large number of dockerins but just a few cohesins like *R. albus and R. bromii*. In *R. albus*, it is likely that a single cohesin serves to anchor isolated dockerin-bearing enzymes onto the cell surface rather than to build a bona fide cellulosome. The starch-degrading enzymes of *R. bromii* bear dockerin domains that enable them to assemble into cohesin-based amylosomes^31^, analogous to cellulosomes, which are active against particulate resistant starches. *R. bromii* strains from the human gut microbiota and the rumen encode similar enzyme complements^31^.

#### Fermentation pathways.

Most of what is known about microbial fermentation pathways in the rumen has been derived from measurements of end product fluxes or inferred from pure or mixed cultures of microorganisms *in vitro*, and based on reference metabolic pathways present in non-rumen microbes. The relative participation of particular species in each pathway, or their contribution to end product formation *in vivo*, is poorly characterized. To determine the functional potential of the sequenced species, we used genome information in combination with the published literature to assign bacteria to different metabolic strategies, on the basis of their substrate utilization and production of specific fermentation end products (Supplementary Table 5). The main metabolic pathways and strategies are present in at least one of, or combinations of, the most abundant bacterial and archaeal groups found in the rumen (Fig. 2b); as a result, we now have a better understanding of which pathways are encoded by these groups. The analysis also provides the first information on the contribution made by the abundant but uncharacterized members of the orders *Bacteroidales* and *Clostridiales* to the rumen fermentation. This metabolic scheme provides a framework for the investigation of gene function in these organisms, and the design of strategies that may enable manipulation of rumen fermentation.

#### Gene loss.

**Figure 3:**
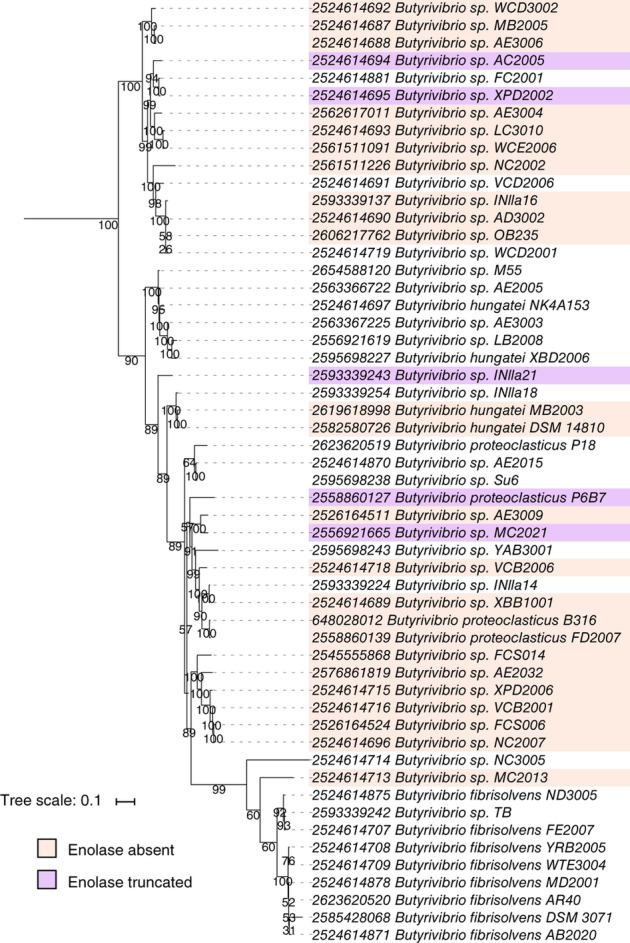
Survey of enolase genes in *Butyrivibrio strains*. Maximum likelihood tree based on concatenated alignment of 56 conserved marker proteins from genomes of all *Butyrivibrio* strains in the Hungate Collection. Strains lacking a detectable enolase gene are indicated by pale pink shading while those with a truncated enolase are indicated by lavender shading. Strains without shading possess an intact enolase.

One curious feature of several rumen bacteria is the absence of an identifiable enolase, the penultimate enzymatic step in glycolysis, which is conserved in all domains of life. Examination of >30,000 isolates from the Integrated Microbial Genomes with Microbiomes (IMG/M) database^32^ revealed that enolase-negative strains were rare (<0.5% of total), and that a high proportion of such strains were rumen isolates belonging to the genera *Butyrivibrio* and *Prevotella* and uncharacterized members of the family *Lachnospiraceae* (Supplementary Table 5). In the genus *Butyrivibrio* approximately half the sequenced strains lack enolase, while some show a truncated form. The distribution of this enzyme in relation to the phylogeny of this genus is shown in Figure 3. This analysis suggests that enolase is in the process of being lost by some rumen *Butyrivibrio* isolates and that we may be observing an example of environment-specific evolution by gene loss^33^. Although the adaptive advantage conferred by loss of enolase is not clear, there is a possible link with pyruvate metabolism and lactate production. Several enolase-negative *Butyrivibrio* strains do not produce lactate and 12 also lack the gene for L-lactate dehydrogenase. Conversely the enolase and L-lactate dehydrogenase genes are co-located in seven strains. An attempt to identify additional functions exhibiting a similar pattern of gene loss (or a complementing gain of function) by comparing enolase-positive versus enolase-negative *Butyrivibrio* spp. strains yielded no substantial additional insights (Supplementary Table 6).

Another example of gene loss is seen in bacteria that have lost their complete glycogen synthesis and utilization pathway, as shown by the concomitant loss of families GH13, GH77, GT3 or GT5, and GT35 (Supplementary Fig. 2). These bacteria include nutritionally fastidious members of the Firmicutes (*Allisonella histaminiformans*, *Denitrobacterium detoxificans*, *Oxobacter pfennigii*) and Proteobacteria (*Wolinella succinogenes*), and have also lost most of their degradative CAZymes, suggesting that they have evolved toward a downstream position as secondary fermenters where they feed on fermentation products (acetate, pyruvate, amino acids) from primary degraders.

#### Biosynthetic gene clusters.

We searched the Hungate genomes for biosynthetic gene clusters (Supplementary Fig. 5 and Supplementary Table 7) to identify evidence of secondary metabolites that might be used as rumen modifiers to reduce methane production through their antimicrobial activity^34^. A total of 6,906 biosynthetic clusters were predicted from the Hungate genomes (Supplementary Note 2).

#### CRISPRs.

Identification of CRISPR–Cas systems and their homologous protospacers from viral, plasmid and microbial genomes could shed light on past encounters with foreign mobile genetic elements^35^ and somewhat indirectly, habitat distribution and ecological interactions^36^. A total of 6,344 CRISPR spacer sequences were predicted from 241 Hungate genomes and searched against various databases (Supplementary Table 8). Searching spacers against a database of cultured and uncultured DNA viruses and retroviruses (IMG/VR) revealed novel associations between 83 viral operational taxonomic units (OTUs) and 31 Hungate hosts. The vast majority of these viruses were derived from human intestinal and ruminal samples. Details and additional results are furnished in Supplementary Note 3.

### Metagenomic sequence recruitment

**Figure 4:**
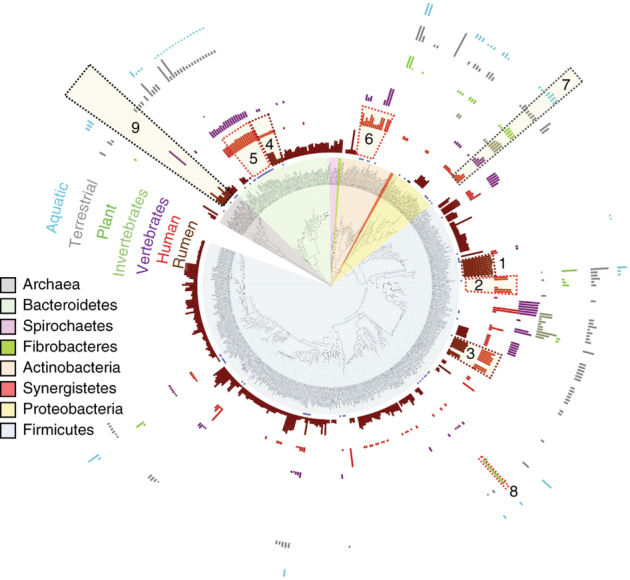
Recruitment of metagenomic proteins by Hungate catalog genomes. Maximum likelihood tree based on 16S rDNA gene alignment of rumen strains. The tree clades are color coded according to phylum. Multi-bar-chart depicting the average % coverage of total CDS of an isolate by metagenome samples from each ecosystem category was drawn using iTOL^55^. Dashed boxes highlight interesting examples of recruitment such as isolates detected in both rumen and human samples (maroon boxes) or detected in human but not rumen samples (red boxes), and others. Number key is as follows (average % coverage is given in parentheses): 1. *Sharpea azabuensis* str. (∼88%), *Kandleria vitulina* str. (∼87%); 2. *Staphylococcus epidermidis* str. (∼40%), *Lactobacillus ruminis* str. (∼51%); 3. *Streptococcus equinus* str. (∼38% by rumen, ∼35% by human); 4. *Prevotella bryantii* str. (∼38% by rumen, ∼9% by human); 5. *Bacteroides* spp.(∼38%); 6. *Bifidobacterium* spp. (∼24%), *Propionibacterium acnes* (∼39%); 7. *Shigella sonnei* (∼30% by human), *E. coli* PA3 (∼31% by human), *Citrobacter* sp. NLAE-zl-C269 (20% by human); 8. *Clostridium beijerinckii* HUN142 (87% by plant); 9. *Methanobrevibacter* spp. (∼32%⋆Figure 4 Maximum likelihood tree based on 16S rDNA gene alignment of rumen strains. The tree clades are color coded according to phylum. Multi-bar-chart depicting the average % coverage of total CDS of an isolate by metagenome samples from each ecosystem category was drawn using iTOL55. Dashed boxes highlight interesting examples of recruitment such as isolates detected in both rumen and human samples (maroon boxes) or detected in human but not rumen samples (red boxes), and others. Number key is as follows (average % coverage is given in parentheses): 1. Sharpea azabuensis str. (~88%), Kandleria vitulina str. (~87%); 2. Staphylococcus epidermidis str. (~40%), Lactobacillus ruminis str. (~51%); 3. Streptococcus equinus str. (~38% by rumen, ~35% by human); 4. Prevotella bryantii str. (~38% by rumen, ~9% by human); 5. Bacteroides spp.(~38%); 6. Bifidobacterium spp. (~24%), Propionibacterium acnes (~39%); 7. Shigella sonnei (~30% by human), E. coli PA3 (~31% by human), Citrobacter sp. NLAE-zl-C269 (20% by human); 8. Clostridium beijerinckii HUN142 (87% by plant); 9. Methanobrevibacter spp. (~32%). The innermost circle identifies Hungate isolates of fecal (★) or salivary (♦) origin. Please refer to Supplementary Table 9 for data and other specifics.

We evaluated whether the Hungate catalog can contribute to metagenomic analyses by using a total of 1,468,357 coding sequences (CDSs) from the 501 reference genomes to search against ∼1.9 billion CDS predicted from more than 8,200 metagenomic data sets from diverse habitats. A total of 892,995 Hungate CDSs (∼60%) were hits to 13,364,644 metagenome proteins at ≥ 90% amino acid identity. 466 out of 501 Hungate isolates recruited sequences from 2,219 metagenomic data sets derived from host-associated, environmental or engineered sources (Fig. 4 and Supplementary Table 9). The large number of human samples recruited (1,699) can be attributed to the greater availability of human samples compared to metagenomes from other mammals, including ruminants. Considering the number of isolate CDSs with hits to metagenome sequences (% coverage), most Hungate genomes (413/501) are represented in rumen metagenome samples, as well as in human or other vertebrate samples (Fig. 4). The average % coverage for 466 recruited genomes was 26.5% of total CDS, with *Sharpea azabuensis* DSM 18934 showing the highest capture (95.6%) in a sheep rumen metagenome (Supplementary Fig. 6).

Examining recruitment against available rumen metagenomes, a majority of 336 isolates were captured in 24 rumen samples (27% average coverage) (Supplementary Fig. 7 and Supplementary Table 9). A further 52 rumen isolates may be included if the hit count recruitment parameter is relaxed from 200 to 50. These isolates are predicted to occur in relatively low abundance in these rumen metagenomes, and raise the proportion of recruiters to almost 80% of the total Hungate catalog. Top recruitment (in terms of % coverage of total isolate CDS) was by organisms previously identified as dominant genera in the rumen^10,22,37,38^, such as *Prevotella* spp., *Ruminococcus* spp., *Butyrivibrio* spp. and members of the unnamed RC-9, R-7 and R-25 groups. Some Hungate catalog genomes were exclusively detected in one or a few samples originating from the same ruminant host (e.g., sheep-associated *Sharpea*, *Kandleria* and *Megasphaera* strains), whereas others were detected across all ruminants (e.g., *Prevotella* spp.). It is, however, important to acknowledge the limitations of existing rumen metagenome samples (not merely in terms of their paucity), as they were sourced from animals on special diets (e.g., switchgrass^5^ or lucerne (alfalfa) pellets^39^), which may alter the microbiome^22^.

165 Hungate cultures were not detected in deposited rumen metagenome data sets under the thresholds applied. Many of these (∼50) were of fecal origin, and reflect how the microbiota of the rumen is distinct from that found in other regions of the ruminant GI tract^40^.

A total of 68 isolates were recruited by both rumen and human intestinal samples and represent shared species between the rumen and human microbiomes (Fig. 4), possibly fulfilling similar roles. A further 66 Hungate isolates were recruited by human samples but were not detected in rumen samples, giving a total of 134 Hungate catalog genomes that recruited various human samples, making them valuable reference sequences for the analysis of human microbiome samples. This observation is also indirectly recapitulated by the CRISPR–CAS systems-based analysis, which showed links to spacers from human intestinal samples, particularly for Hungate isolates of fecal origin (Supplementary Table 8). Additional metagenome recruitment analysis details are provided in Supplementary Note 4.

### Comparison with human gut microbiota

**Figure 5:**
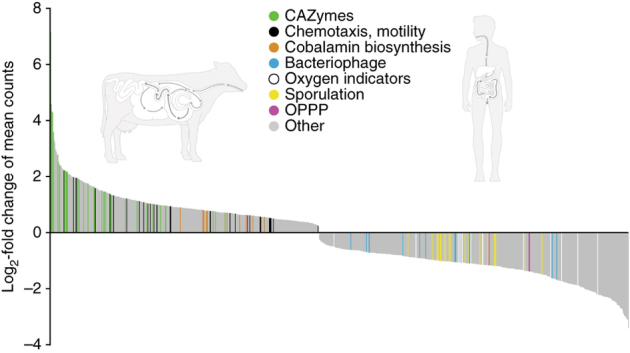
Differentially abundant Pfams between rumen and human intestinal isolates. *X* axis is individual Pfams detected by Metastats to be differentially abundant with a Q-value < 0.001. *Y* axis is the log2-fold difference of mean counts for each population (rumen or intestinal). Select Pfams are highlighted as discussed in the text. OPPP, oxidative pentose phosphate pathway.

Many Hungate strains (134/501) were shared between rumen and human intestinal microbiome samples. This is unsurprising, as both habitats are high-density, complex anaerobic microbial communities, producing similar fermentation products, and with extensive interspecies cross-feeding and interaction^41^. We performed a comparative analysis against available human intestinal isolates (largely from the HMP), to identify differences that can be attributed to distinct lifestyles and adaptive capacity of rumen microorganisms. The Hungate and human intestine isolate collections were curated to remove redundancy, low-quality genomes and known human pathogens. This resulted in a set of 458 rumen and 387 human intestinal genomes (Supplementary Table 10), which was used to identify protein families in the Pfam database that were differentially abundant in isolates from each environment. Out of 7,718 Pfam domains found in 458 non-redundant Hungate isolate genomes, we determined 367 were over-represented in the ruminal genomes and 423 were under-represented on the basis of the false-discovery rate (FDR), q-value < 0.001 (Supplementary Table 11). Over-represented Pfams (Fig. 5) included enzymes involved in plant cell wall degradation (GH11, GH16, GH26, GH43, GH53, GH67, GH115), carbohydrate-binding modules (CBM2, CBM3, and cohesin and dockerin modules associated with cellulosome assembly) and GT41 family glycosyl transferases, which occur predominantly in the genera *Anaerovibrio* and *Selenomonas*. Notably, Pfams for the biosynthesis of cobalamin (vitamin B_12_, an essential micronutrient for the host, were over-represented. Vitamin B_12_ biosynthesis is one of the most complex pathways in nature, involving more than 30 enzymatic steps, and given its high metabolic cost, is only encoded by a small set of bacteria and archaea. We examined this biosynthetic pathway in more detail using other functional annotation types (KO and Tigrfam) across the 501 Hungate isolates, and discovered that 12 or more enzymatic steps were overrepresented in the Hungate genomes, and at least 47 isolates might be capable of *de novo* B_12_ synthesis (Supplementary Table 12). Many of these were members of the Class *Negativicutes* within the Firmicutes (*Anaerovibrio*, *Mitsuokella*, and *Selenomonas*). A further 140 (including 21 archaeal) genomes encode enzymes for the salvage of B_12_ from an intermediate, and may even work cooperatively (based on potential complementarity of lesions in the pathway in different members) to share and synthesize corrinoids for community and/or host benefit. These observations reflect the high burden of a requirement for vitamin B_12_, which is needed as a cofactor for enzymes involved in gluconeogenesis from propionate in the liver. This process is essential for lactose biosynthesis and milk production in dairy animals^42^, and dairy and meat products of ruminant origin are important dietary sources of B_12_ (ref. 43). By contrast, it has been speculated that human gut microbes were unlikely to contribute significant amounts of B_12_ for their host and were likely competitors for dietary B_12_ (ref. 44).

Of the Pfams (Fig. 5) under-represented in Hungate genomes, the occurrence of all steps for the oxidative branch of the pentose phosphate pathway (OPPP) was striking. The role of the OPPP is primarily the irreversible production of reducing equivalents (NADPH), although other enzymes may serve as alternate sources of reducing equivalents. As discussed above, the Pfam for enolase appeared in the list of under-represented families. The list also contained several Pfams associated with bacteriophage functions and sporulation. The differential abundance of sporulation genes is interesting as the observation that sporulation genes are abundant in human gut bacteria has been made recently^16,31^ and is potentially linked with resistance to oxygen exposure. This observation is particularly striking given the preponderance of Firmicutes, an archetypically spore-forming phylum^45^, in the rumen set. Large and small subunits of an oxygen-dependent Class I type ribonucleotide reductase were also under-represented together with several other Pfams implicated in oxygen tolerance, suggesting that human intestinal isolates may encounter higher oxygen tension compared to the strictly anaerobic ruminal ecosystem. These observations indirectly suggest that host genetics and physiology influence rumen microbiome composition and that rumen microbes are likely to be vertically inherited as indicated in recent studies^46,47^. Conversely, human intestinal (more specifically, fecal) isolates are transmitted from other sources in the environment^31,48^. We were able to recapitulate these findings in a metagenome-based comparison of these two environments (sheep rumen samples against normal human fecal samples; Supplementary Table 13), suggesting that these differences cannot be explained by cultivation or abundance biases in the isolate data sets.

## Discussion

The Hungate genome catalog that we report here includes genomic analysis of 501 bacterial and archaeal cultures that represent almost all of the cultured rumen species that have been taxonomically characterized, as well as representatives of several novel species and genera. This high-quality reference collection will guide interpretation of metagenomics data sets, including genomes recovered from metagenomes (MAGs). The Hungate genome catalog also allows robust comparative genomic analyses that are not feasible using incomplete sequence data from metagenomes. Researchers have access to Hungate Collection strains, which will enable a better understanding of carbon flow in the rumen, including the breakdown of lignocellulose, through the metabolism of substrates to SCFAs and fermentation end products, to the final step of CH_4_ formation.

The Hungate genome collection is by no means complete. Some important taxa are missing, especially members of the order *Bacteroidales*^10,22^. At the start of this project genome sequences were available for strains belonging to 11 (12.5%) of the 88 genera described for the rumen. Currently, genome sequences are available for 73 (83%) of those 88 genera, as well as for 73 strains that are only identified to the family or order taxonomic level. Of the rumen 'most wanted list' which comprises 70 rumen bacteria^10^, the Hungate Collection has now contributed 30 members. In addition to missing bacteria and archaea, the sequencing of rumen eukaryotes presents considerable technical challenges and although some progress has been made in sequencing of anaerobic fungi^49^, there are no genome data for rumen ciliate protozoa, and only preliminary data on the rumen virome^50^.

Microbiome research is moving from descriptive to mechanistic, and to translation of those mechanisms into interventions^51^. Using rumen microbiome data to engineer rumens to reduce CH_4_ emissions^52^ and improve productivity and sustainability outcomes is now in sight^53^. The Hungate Collection provides a starting point for this, shedding light on what has been described as 'the world's largest commercial fermentation process'^54^. Future studies can use the Hungate resources to improve the resolution of rumen meta-omics analyses, to identify antimicrobials, to source carbohydrate-degrading enzymes from the rumen for use as animal feed additives and in lignocellulose-based biofuel generation, and as the basis for synthetic microbial consortia.

## Methods

### Cultures used in this study.

The full list of cultures used in the project and their provenance is shown in Supplementary Table 1 with additional information available in Supplementary Note 1. New Zealand bacterial cultures from the Hungate Collection are available from the AgResearch culture collection while other cultures should be obtained from the relevant culture collections or requested from the sources shown in Supplementary Table 1.

### Genomic DNA isolation.

Genomic DNA was extracted using the Qiagen Genomic-tip kit following the manufacturer's instructions for the 500/G size extraction. Purified DNA was subject to partial 16S rRNA gene sequencing to confirm strain identity, before being shipped to the DOE Joint Genome Institute (JGI), USA for sequencing.

### Sequence, assembly and annotation.

All Hungate genomes were sequenced at the DOE Joint Genome Institute (JGI) using Illumina technology^56^ or Pacific Biosciences (PacBio) RS technology^57^. For all genomes, we either constructed and sequenced an Illumina short-insert paired-end library with an average insert size of 270 bp, or a Pacbio SMRTbell library. Genomes were assembled using Velvet^58^, ALLPATHS^59^ or Hierarchical Genome Assembly Process (HGAP)^60^ assembly methods (specifics provided in Supplementary Table 2). Genomes were annotated by the DOE–JGI genome annotation pipeline^61,62^. Briefly, protein-coding genes (CDSs) were identified using Prodigal^63^ followed by a round of automated and manual curation using the JGI GenePrimp pipeline^64^. Functional annotation and additional analyses were performed within the Integrated Microbial Genomes (IMG-ER) platform^32^. All data as well as detailed sequencing and assembly reports can be downloaded from https://genome.jgi.doe.gov/portal/pages/dynamicOrganismDownload.jsf?organism=HungateCollection.

### Hungate Collection and the Global Rumen Census analysis.

We used the 16S rRNA gene sequences generated from the Global Rumen Census (GRC)^22^ to map the phylogenetic positions of the Hungate Collection genomes onto the known global distribution of Bacteria and Archaea from the rumen. Ten-thousand predicted OTUs were randomly chosen from the total 673,507 OTUs identified from that study in order to construct a phylogenetic tree. The 16S rRNA gene sequences for Hungate Collection genomes were added to the GRC subsample, and all Bacteria and Archaea were checked for chimeras and to ensure they represented separate OTUs using CDHIT-OTU^65^ (with a 0.97% identity Ribosomal Database Project (RDP)^66^ followed by visual inspection with JalView^67^. Taxonomic classifications were taken from those predicted by the GRC study. A maximum likelihood tree was then separately constructed for the Bacteria and Archaea using two rounds of Fasttree (version 2.1.7)^68^: the first round built a maximum likelihood tree using the GTR model of evolution and (options: -gtr –nt); the second round optimized the branch lengths for the resulting topology (options: -gtr -nt -nome –mllen). The resulting phylogenetic trees were visualized using iTOL^55^ with the mapped positions of the Hungate genomes.

### Carbohydrate-active enzymes (CAZymes).

For each of the 501 genomes, the protein sequences were subjected to parallel (i) BLAST queries against CAZy libraries, of both complete sequences and individual modules; and (ii) HMMER searches using CAZy libraries of module family and subfamilies. Family assignments and overall CAZyme modularity were further validated through a human curation step, when proteins were not fully aligned (without gaps) with >50% identity to CAZy records.

### Conserved single-copy gene phylogeny.

A set of 56 universally conserved single-copy proteins in bacteria and archaea^69^ was used for construction of the *Butyrivibrio* phylogenetic tree. Marker genes were detected and aligned using hmmsearch and hmmalign included in HMMER3 (ref. 70) using HMM profiles obtained from Phylosift^71^. Alignments were concatenated and filtered. A phylogenetic tree was inferred using the maximum likelihood methods with RAxML (version 7.6.3). Tree topologies were tested for robustness using 100 bootstrap replicates and the standard LG model. Trees were visualized using FastTree followed by iTOL^55^.

### Prediction of biosynthetic clusters.

Putative biosynthetic clusters (BCs) were predicted and annotated using AntiSMASH version 3.0.4 (ref. 72) with the “inclusive” and the “borderpredict” options. All other options were left as default.

### CRISPR–CAS system analysis.

A modified version of the Crispr Recognition Tool (CRT) algorithm^61^, with annotations from the Integrated Microbial Genomes with Metagenomes (IMG/M) system^32^ was used to validate the functionality of the CRISPR–Cas types (only complete cas gene arrangements were used plus those cas 'orphan' arrays with the same repeat from a complete array within the same genome). This Hungate spacer collection was queried against the viral database from the Integrated Microbial Genome system (IMG/VR database)^73^, a custom global “spacerome” (predicted from all IMG isolate and metagenome data sets) and the NBCI refseq plasmid database. All spacer searches were performed using the BLASTn-short function from the BLAST+ package^74^ with parameters: e-value threshold of 1.0 × 10^−6^, percentage identity of >94% and coverage of >95%. These cutoffs were recommended by a recent study benchmarking the accuracy of spacer hits across a range of % identities and coverage^75^.

### Recruitment of metagenomic sequences.

1,468,357 protein coding sequences or CDS from 501 Hungate isolate genomes were searched using LAST^76^ against ∼1.9 billion CDS predicted from 8,200 metagenomic samples stored in the IMG database. Hungate genomes were designated as “recruiters” if the following criteria were met: a minimum of 200 CDS with hits at ≥ 90% amino acid identity over 70% alignment lengths to an individual metagenomic CDS or ≥ 10% capture of total CDS in each genome. The rationale for choosing the minimum 200 hit count was to ensure that the evidence included more than merely housekeeping genes (which tend to be more highly conserved). In a few instances, the 200 CDS hit count requirement was relaxed if at least 10% of the total CDS in the genomes was captured. The 90% amino acid identity cutoff was chosen based on Luo *et al*.^77^, who assert that organisms grouped at the 'species' level typically show >85% AAI among themselves. We ascertained that ≥ 90% identity was sufficiently discriminatory for species in the Hungate genome set by observing differences in the recruitment pattern (hit count or % CDS coverage) of different species of the same genus (e.g., *Prevotella spp*., *Butyrivibrio* spp., *Bifidobacterium* spp., *Treponema* spp.) from every phylum against the same metagenomic sample.

For nucleotide read recruitment, total reads from an individual metagenome were aligned against scaffolds from each of the 501 isolates using the BWA aligner^78^. The effective minimum nucleotide % identity was ∼75% with a minimum alignment length of 50 bp. Alignment results were examined in terms of total number of reads recruited to an isolate (at different % identity cutoffs with ≥ 97% identity proposed as a species-level recruitment), average read depth of total reads recruited to a given isolate genome, as well as % coverage of total nucleotide length of the genome.

### Genome comparisons.

For rumen versus human isolates comparisons, human intestinal isolate genomes were carefully selected from the IMG database using available GOLD metadata fields pertaining to isolation source (and taking care to remove known pathogens). Genome redundancies within either the human set or the rumen set were eliminated after assessing the average nucleotide identity (ANI) of total best bidirectional hits and removing genomes sharing >99% ANI (alignment fraction of total CDS ≥ 60%) to another genome within that set. Furthermore, low-quality genomes within the human set were flagged and removed based on the absence of the “high-quality” filter assigned by the IMG quality control pipeline owing to lack of phylum-level taxonomic assignment or if the coding density was <70% or >100% or the number of genes per million base pairs was <300 or >1,200 (ref. 61). This approach resulted in 388 genomes delineated in the human set and 458 genomes in the rumen set (lists provided in Supplementary Table 10). Both collections of genomes had similar average genome sizes (3.3–3.5 Mbp) and completeness (evaluated by CheckM^19^). Pairwise comparisons of gene counts for individual Pfams between members of each set were performed using Metastats^79^, which employs a non-parametric two-sided *t*-test test (or a Fischer's exact test for sparse counts) with false-discovery rate (FDR) error correction to identify differentially abundant features between the two genome sets. Most significant features were delineated using a q-value cutoff of <0.001, and less populous or sparsely recruited Pfams were also eliminated (where the sum of gene counts in each genome set was <100) (Supplementary Table 11, worksheet designated “Q-val<0.001_edited”). A second worksheet labeled “Q-val<0.005” shows a larger subset of differentially abundant Pfams applying the less stringent threshold of Q-value < 0.005, and including results for Pfams with sparse counts. Pfam was chosen for this primary analysis because it is the largest and most widely used source of manually curated protein families, with nearly 80% coverage (on average) of total CDS in these microbial genomes. KO terms or TIGRFAMS were also assessed to validate and complement Pfam-based findings or to examine specific pathways more closely. For comparisons of enolase-positive versus enolase-negative *Butyrivibrio* spp. strains, Metastats^79^ was employed in conjunction with contrasting upper and lower quartile or percentile gene counts, in order to identify additional functions with a similar pattern of preservation/loss as the glycolytic enolase gene.

For metagenomes-based comparisons, previously published sheep rumen (IMG IDs: 3300021254, 300021255, 3300021256, 3300021387, 3300021399, 3300021400, 3300021426, 3300021431) and human intestinal (IMG IDs: 3300008260, 3300008496, 3300007299, 3300007296, 3300008272, 3300007361, 3300008551, 3300007305, 3300007717) metagenomes were reassembled using metaSPAdes^80^, annotated and loaded into IMG. Estimated gene copy numbers (calculated by multiplying gene count with read depth for the scaffold the gene resides on) were compared using Metastats (as described above).

### Statistical analysis.

Refer to the Life Sciences Reporting Summary.

### Life Sciences Reporting Summary.

Further information on experimental design is available in the Life Sciences Reporting Summary.

### Data availability.

All available genomic data and annotations are available through the IMG portal (https://img.jgi.doe.gov/). Additionally, a dedicated portal to download all 410 genomes sequenced in this study is provided: https://genome.jgi.doe.gov/portal/pages/dynamicOrganismDownload.jsf?organism=HungateCollection.

## Additional information

**Publisher's note:** Springer Nature remains neutral with regard to jurisdictional claims in published maps and institutional affiliations.

## Supplementary Information

### Integrated supplementary information

Supplementary Figure 1 Phylum and genome size distribution of the 501 Hungate catalogue genomes. a) Phylum distribution of the sequenced organisms.
b) Genome size distribution in the different phyla of sequenced organisms.

Supplementary Figure 2 Carbohydrate degradative capabilities of the 501 Hungate catalogue genomes. Family counts of degradative CAZymes (glycoside hydrolases (GHs) and polysaccharide lyases (PLs)), dockerins (DOC) and cohesins (COH) were used to cluster the 501 Hungate genomes. Hierarchical clustering was realized with Spearman’s rank correlation as distance and pairwise average-linkage as clustering method. Phyla with six or less representative species (grouped as "Others") and main taxonomic phyla were distinguished by a colour-code at left. Clusters of CAZyme families involved in the breakdown of selected polysaccharides are coloured at the bottom. Coloured boxes outline specific taxonomic groups that degrade these polysaccharides. Arrows indicate the rare absence of the GH13 starch/glycogen family.

Supplementary Figure 3 The most abundant CAZyme (glycoside hydrolase) families among 501 Hungate catalogue genomes. Dark green, plant structural carbohydrates (cellulose, hemicellulose, pectin); Light green, plant storage carbohydrates (fructans, raffinose, starch); blue, peptidoglycan.

Supplementary Figure 4 Genomic view of the PUL for rhamnogalacturonan type II breakdown in B. thetaiotaomicron VPI-5482 and microsyntenic regions in the species studied in this work. Protein-coding genes are depicted by coloured rectangles to highlight the following functional modules: GHs in light pink, PLs in dark pink, HTCS and ECF-σ/anti-σ factor regulators in cyan, MFS and SusC transporters in purple, SusD outer membrane proteins in orange, peptidases in gold, esterases (Ac for acetyl and Me for methyl) in brown. Polygons, coloured according to functional modules, outline the orthologs and reveal likely genomic rearrangements between species. Triangles with dotted-lines indicate specific insertion/deletion between highly similar regions. Genes are represented either above or below a central black line to represent the coding strand. When PUL genes are split across several scaffolds, due to incomplete genome assembly, the scaffold limits are indicated by vertical red bars.

Supplementary Figure 5 Distribution of genes encoding antimicrobial biosynthetic clusters (bacteriocins, lantipeptides and non-ribosomal peptide synthases) in the Hungate catalogue genomes Maximum likelihood tree based on 16S rDNA gene alignment was visualized and annotated using iTOL. Tree clades are colour coded according to phylum. Multi-bar-charts depict the total number of biosynthetic clusters for putative antimicrobial secondary metabolite classes in each genome.

Supplementary Figure 6 Protein recruitment plot showing amino acid % identity (y-axis) of top hits of *Sharpea azabuensis* DSM 18934 CDSs against metagenomic sequences from New Zealand sheep rumen samples. Isolate CDSs are ordered on the x-axis by position on individual scaffolds (which are themselves ordered by descending sequence length) available for this genome.

Supplementary Figure 7 Recruitment of rumen metagenomes by Hungate catalogue genomes. Maximum likelihood tree based on 16S rDNA gene alignment was visualized and annotated using iTOL. Tree clades are colour coded according to phylum. Bar-charts depict the average % coverage of total CDSs of an isolate by rumen metagenome samples from each ruminant host shown on individual circles around the tree.

### Supplementary information

Supplementary Text and Figures Supplementary Figures 1–7 (PDF 1301 kb)

Life Sciences Reporting Summary (PDF 129 kb)

Supplementary Table 1 Cultures and their provenance. (XLSX 46 kb)

Supplementary Table 2 Genome statistics for the 410 sequenced bacteria and archaea. (XLSX 66 kb)

Supplementary Table 3 Genome sequencing projects for rumen microbes. (XLSX 14 kb)

Supplementary Table 4 CAZyme analysis. GH, glycoside hydrolases; PL, polysaccharide lyases;∼GT, glycosyl transferases; DOC, dockerins, COH, cohesins; CE, carbohydrate esterases; CBM, carbohydrate-binding modules; AA, auxillary activities. (XLSX 505 kb)

Supplementary Table 5 Metabolic strategies and metabolic pathways encoded by the Hungate catalogue genomes. (XLSX 42 kb)

Supplementary Table 6 List of differentially abundant Pfams in enolase-positive versus enolasenegative strains of Butyrivibrio spp. (XLSX 244 kb)

Supplementary Table 7 Biosynthetic clusters identified in the Hungate catalogue genomes. (XLSX 408 kb)

Supplementary Table 8 Hungate CRISPR spacer search results (XLSX 596 kb)

Supplementary Table 9 Protein recruitment results of rumen isolate CDS by individual metagenomes in IMG. (XLSX 1131 kb)

Supplementary Table 10 List of 458 rumen isolates and 387 human intestinal isolates used for genome comparisons. (XLSX 44 kb)

Supplementary Table 11 List of Pfams that are over-represented (green highlight) or underrepresented (pink highlight) in the ruminal compared to the human intestinal isolate genomes. (XLSX 386 kb)

Supplementary Table 12 Occurrence of vitamin B12 biosynthetic pathway genes in the rumen isolate genomes. Green highlight indicates genomes with predicted de novo biosynthetic capability. (XLSX 89 kb)

Supplementary Table 13 List of Pfams that are over-represented (green highlight) or underrepresented (pink highlight) in the sheep ruminal metagenomes compared to the human intestinal metagenomes. (XLSX 1118 kb)

Supplementary Notes 1–4 (PDF 1162 kb)
