# Supplementary material for: Cultivation and sequencing of rumen microbiome members from the Hungate1000 Collection
Source: Nat Biotechnol. 2018 Mar 19;36(4):359–67. doi: 10.1038/nbt.4110 (PMC6118326; doi:10.1038/nbt.4110)
Supplement: Supplementary Text and Figures — Supplementary Figures 1–7 (PDF 1301 kb) [file 41587_2018_BFnbt4110_MOESM6_ESM.pdf]

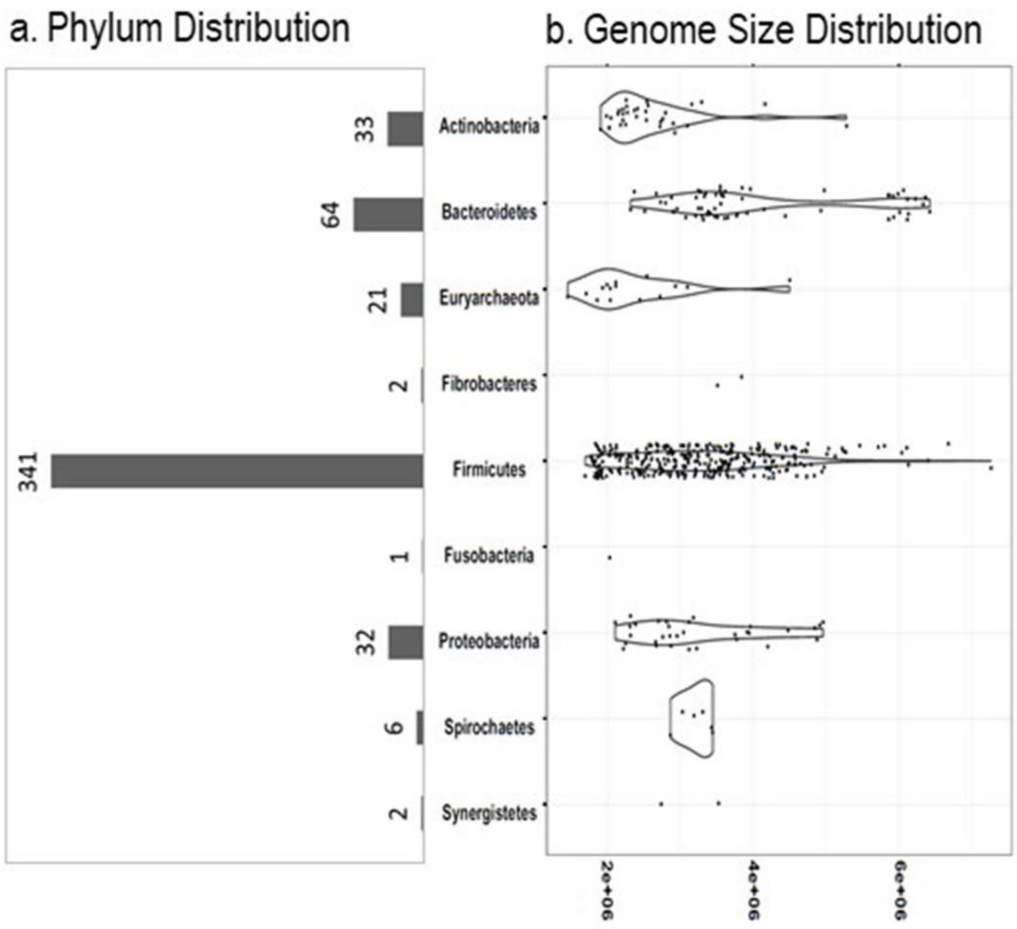

Supplementary Figure 1

Phylum and genome size distribution of the 501 Hungate catalogue genomes.

a) Phylum distribution of the sequenced organisms.

b) Genome size distribution in the different phyla of sequenced organisms.

Nature Biotechnology: doi:10.1038/nbt.4110

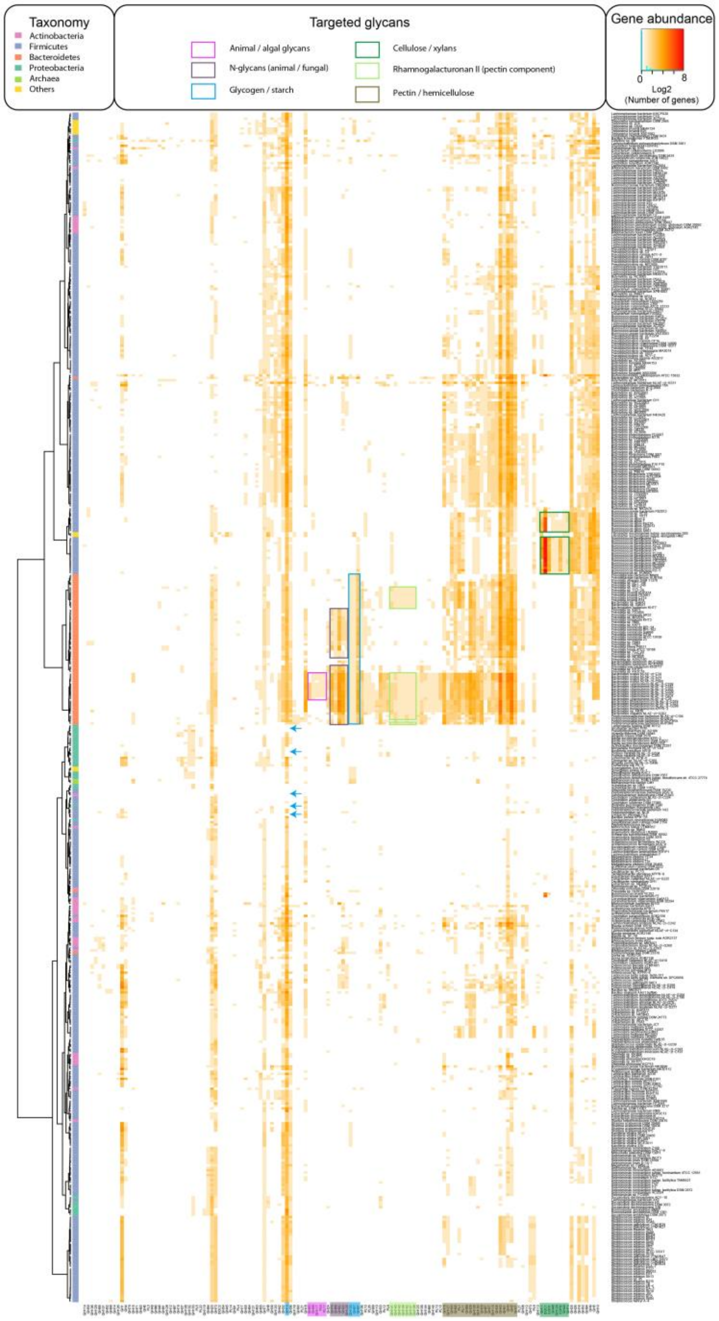

Supplementary Figure 2

Carbohydrate degradative capabilities of the 501 Hungate catalogue genomes.

Family counts of degradative CAZymes (glycoside hydrolases (GHs) and polysaccharide lyases (PLs)), dockerins (DOC) and cohesins (COH) were used to cluster the 501 Hungate genomes. Hierarchical clustering was realized with Spearman's rank correlation as distance and pairwise average-linkage as clustering method. Phyla with six or less representative species (grouped as "Others") and main taxonomic phyla were distinguished by a colour-code at left. Clusters of CAZyme families involved in the breakdown of selected polysaccharides are coloured at the bottom. Coloured boxes outline specific taxonomic groups that degrade these polysaccharides. Arrows indicate the rare absence of the GH13 starch/glycogen family.

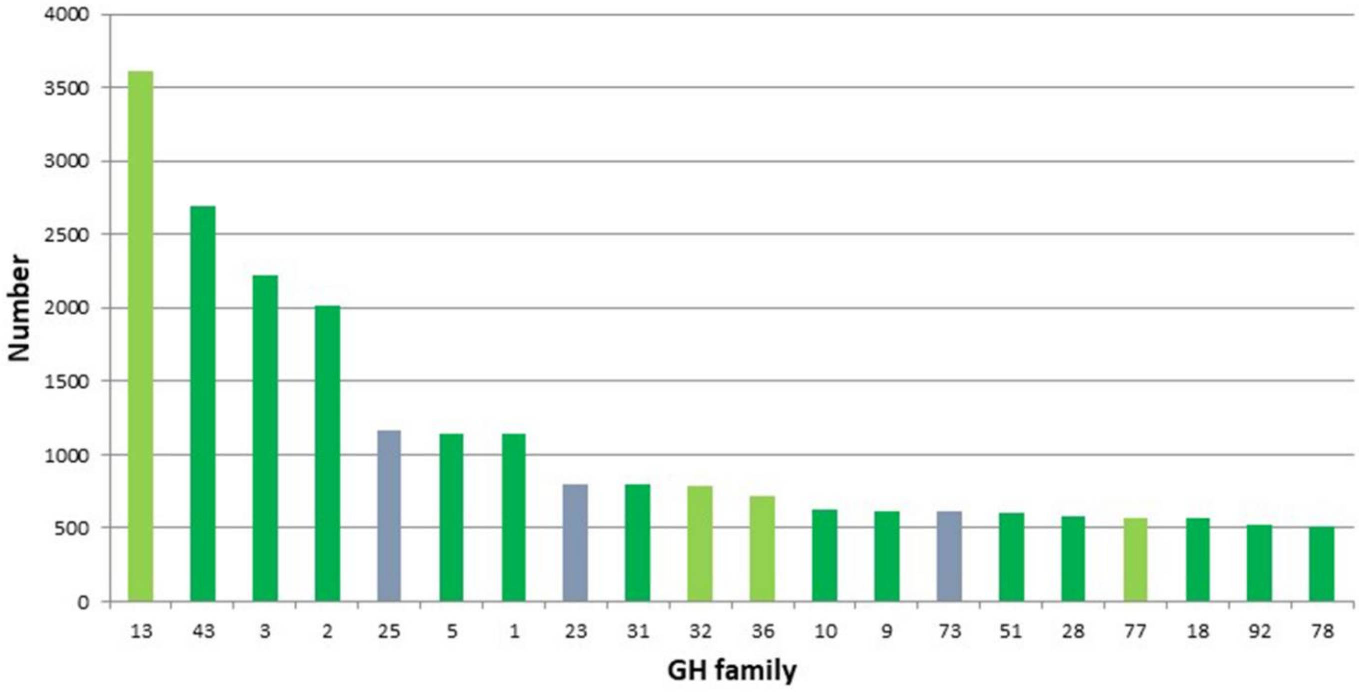

Supplementary Figure 3

The most abundant CAZyme (glycoside hydrolase) families among 501 Hungate catalogue genomes.

Dark green, plant structural carbohydrates (cellulose, hemicellulose, pectin); Light green, plant storage carbohydrates (fructans, raffinose, starch); blue, peptidoglycan.

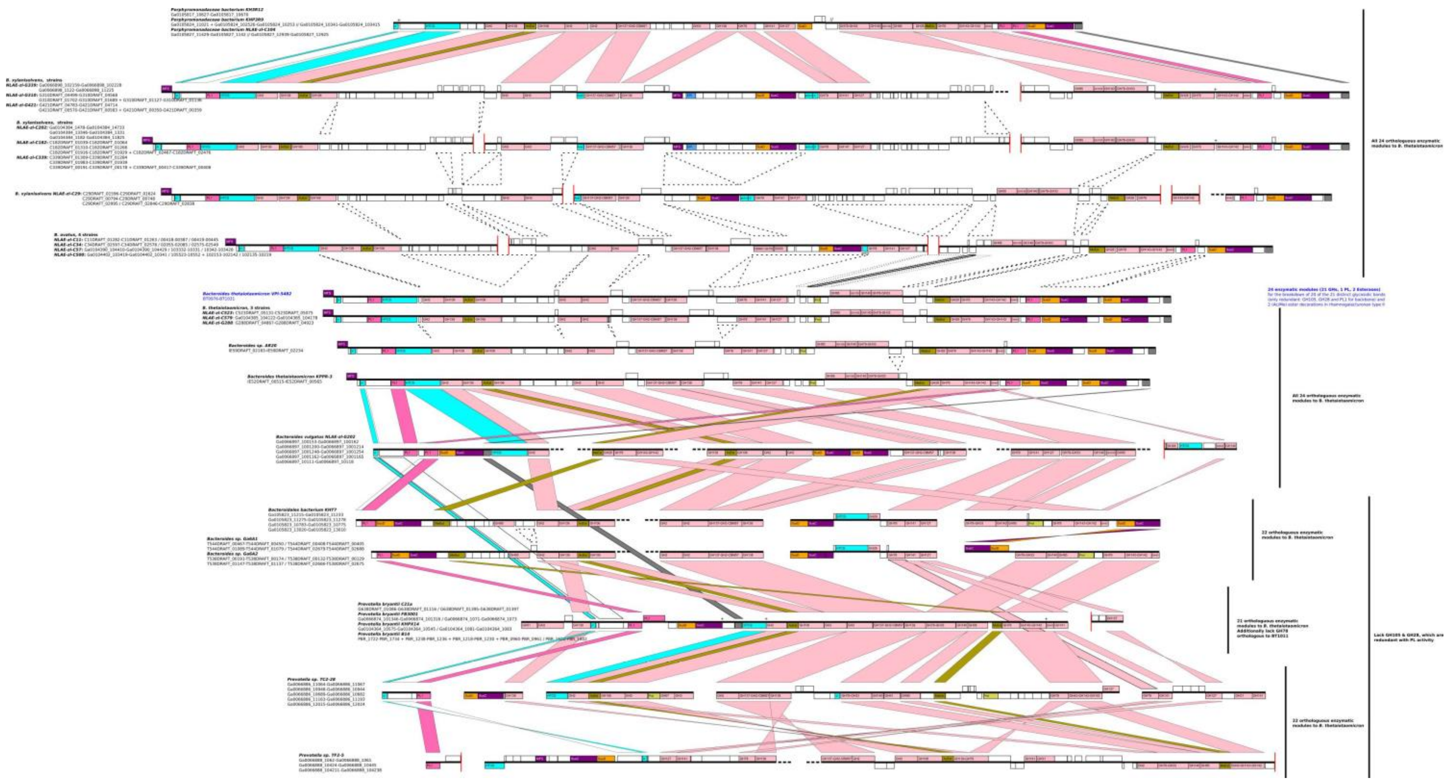

Supplementary Figure 4

Genomic view of the PUL for rhamnogalacturonan type II breakdown in B. thetaiotaomicron VPI-5482 and microsyntenic regions in the species studied in this work.

Protein-coding genes are depicted by coloured rectangles to highlight the following functional modules: GHs in light pink, PLs in dark pink, HTCS and ECF-σ/anti-σ factor regulators in cyan, MFS and SusC transporters in purple, SusD outer membrane proteins in orange, peptidases in gold, esterases (Ac for acetyl and Me for methyl) in brown. Polygons, coloured according to functional modules, outline the orthologs and reveal likely genomic rearrangements between species. Triangles with dotted-lines indicate specific insertion/deletion between highly similar regions. Genes are represented either above or below a central black line to represent the coding strand. When PUL genes are split across several scaffolds, due to incomplete genome assembly, the scaffold limits are indicated by vertical red bars.

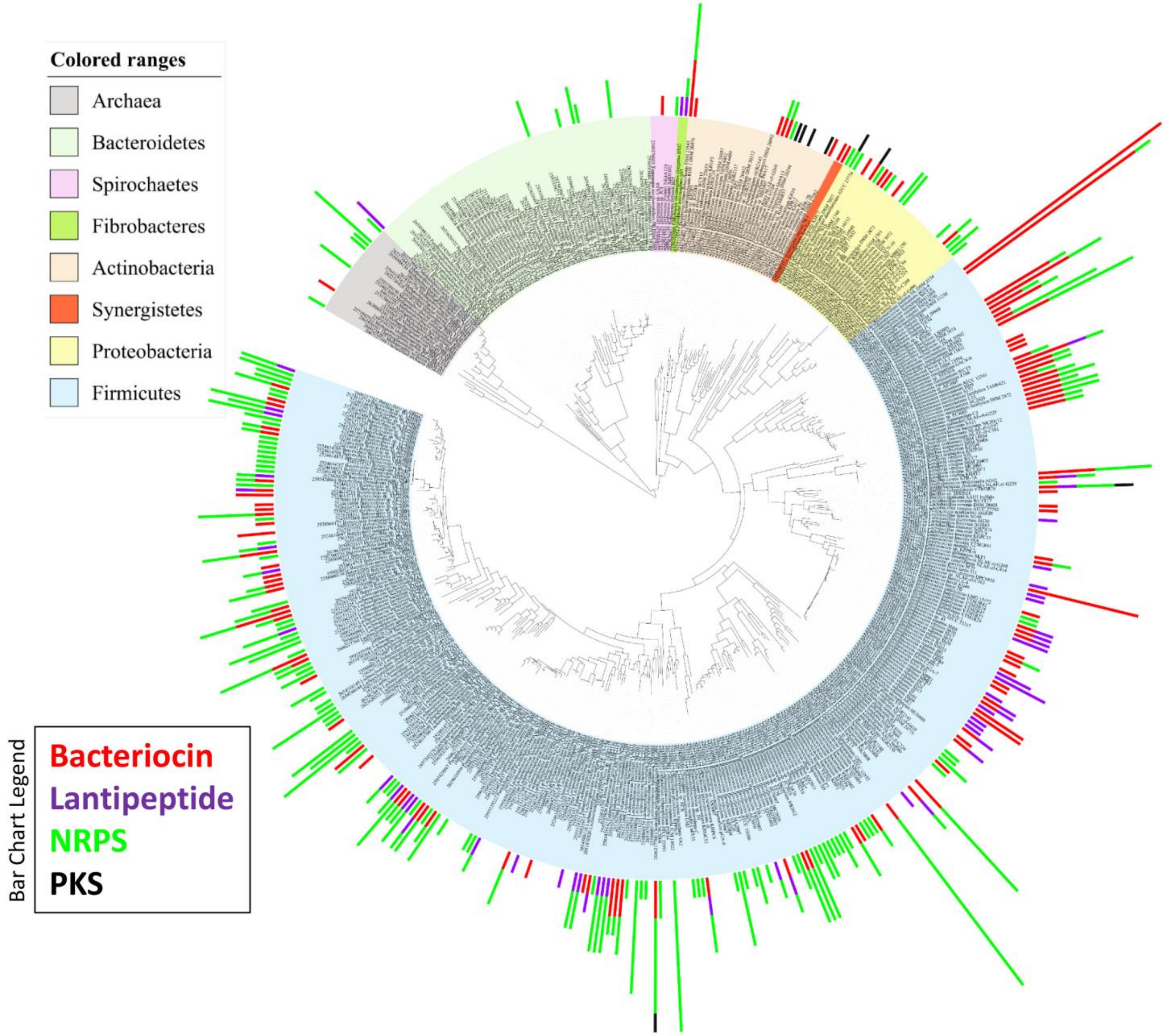

Supplementary Figure 5

Distribution of genes encoding antimicrobial biosynthetic clusters (bacteriocins, lantipeptides and non-ribosomal peptide synthases) in the Hungate catalogue genomes

Maximum likelihood tree based on 16S rDNA gene alignment was visualized and annotated using iTOL. Tree clades are colour coded according to phylum. Multi-bar-charts depict the total number of biosynthetic clusters for putative antimicrobial secondary metabolite classes in each genome.

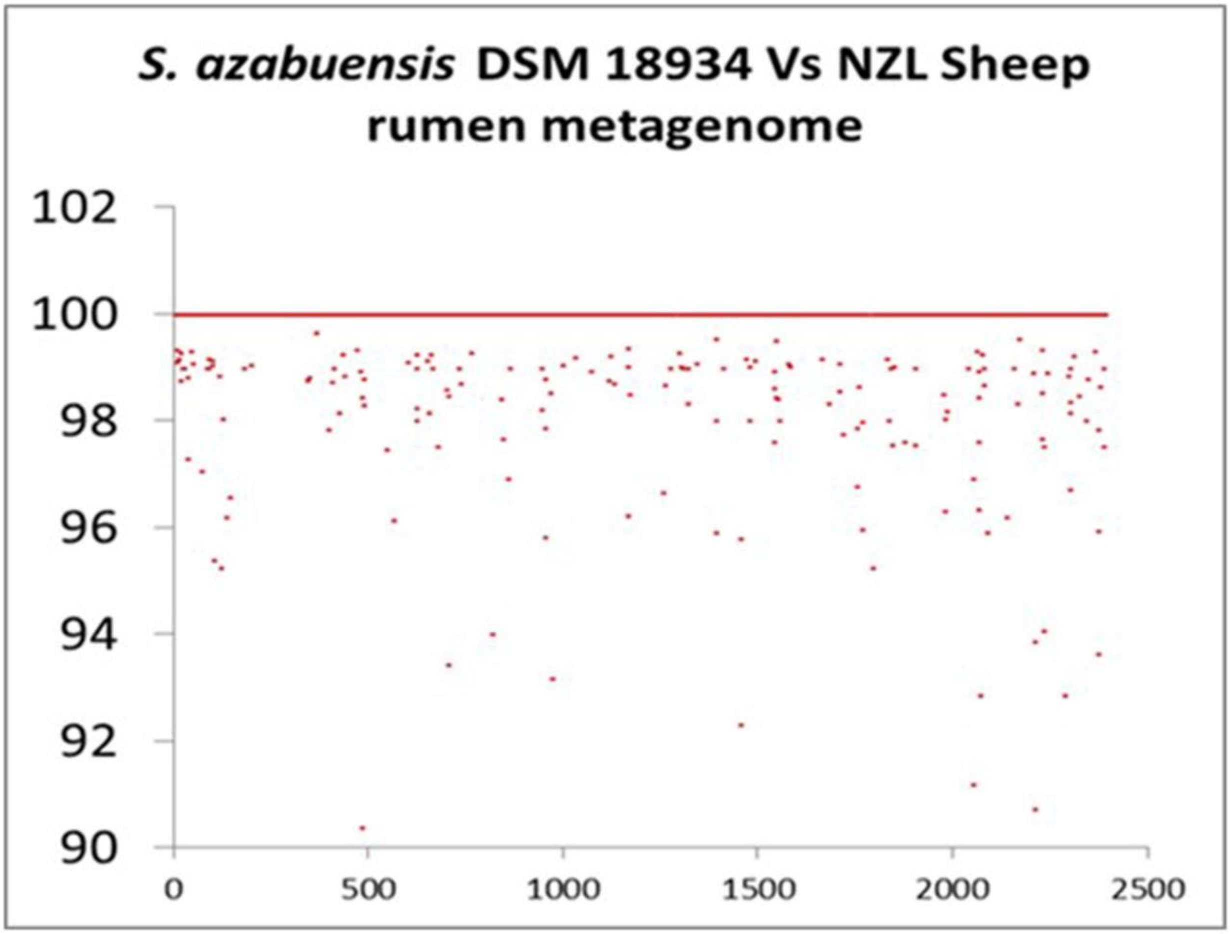

Supplementary Figure 6

Protein recruitment plot showing amino acid % identity (y-axis) of top hits of *Sharpea azabuensis* DSM 18934 CDSs against metagenomic sequences from New Zealand sheep rumen samples.

Isolate CDSs are ordered on the x-axis by position on individual scaffolds (which are themselves ordered by descending sequence length) available for this genome.

Nature Biotechnology: doi:10.1038/nbt.4110

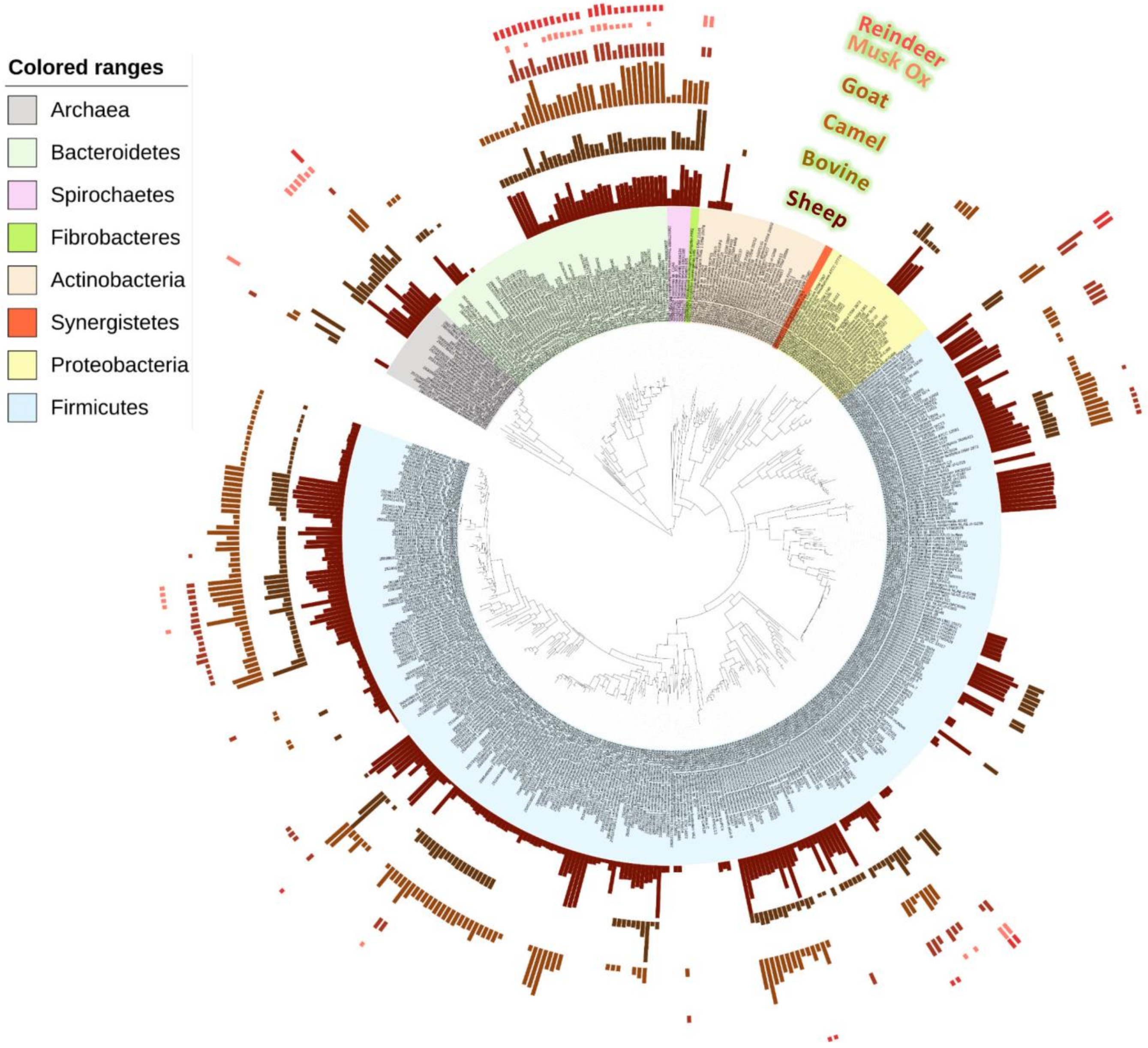

Supplementary Figure 7

Recruitment of rumen metagenomes by Hungate catalogue genomes.

Maximum likelihood tree based on 16S rDNA gene alignment was visualized and annotated using iTOL. Tree clades are colour coded according to phylum. Bar-charts depict the average % coverage of total CDSs of an isolate by rumen metagenome samples from each ruminant host shown on individual circles around the tree.
