# Supplementary material for: Cultivation and sequencing of rumen microbiome members from the Hungate1000 Collection
Source: Nat Biotechnol. 2018 Mar 19;36(4):359–67. doi: 10.1038/nbt.4110 (PMC6118326; doi:10.1038/nbt.4110)
Supplement: Supplementary file 23 — Supplementary Notes 1–4 (PDF 1162 kb) [file 41587_2018_BFnbt4110_MOESM21_ESM.pdf]

SUPPLEMENTARY NOTE 1

Cultures used in this study

The primary aim of the Hungate1000 project was to generate a reference set of rumen microbial genome sequences. The first step in this process was to compile a list of cultured rumen isolates in order to determine the range of cultures available. This identified 146 rumen isolates held in culture collections, and reports of 88 different genera belonging to nine phyla[160]. The collection of rumen archaea and bacteria held at the AgResearch Grasslands Research Centre served as a starting point for this project, and members of the Rumen Microbial Genomics Network (http://www.rmgnetwork.org/) were surveyed and requested to provide cultures of interest. These were supplemented with additional cultures purchased from culture collections. The full list of cultures used in the project and their provenance is shown in Supplementary Table 1.

The largest single group of bacterial cultures (113) used in this project was isolated from the fibre-adherent fraction of bovine rumen contents during a companion study designed to bring additional rumen bacteria into cultivation[8]. In this study rumen contents were sampled from five ruminally fistulated Holstein/Friesian dairy cows at five time points over the course of a year (four animals at each time point). The animals, feed, rumen sampling methods and the protocol used to separate digesta-adherent bacteria have been described previously[161]. Bacteria were isolated from the digesta-adherent fraction using the techniques and media described by Kenters *et al*[25]. Briefly, the plant-adherent fraction was obtained by washing the rumen digesta four times with basal RM02 medium[25], followed by squeezing through cheesecloth. The retained fraction was then blended to release the bacterial cells from the plant material. All of these steps were done anaerobically under a stream of CO_2. Serial dilutions of the blended digesta were made in order to obtain a range around a theoretical number of 0.2 viable cells/mL. Serial 10- and 100-fold dilutions were made down to 10^{-7}-

Nature Biotechnology: doi:10.1038/nbt.4110

fold. This dilution was then further diluted in a series of six four-fold dilutions. All dilutions were made in RM02 base media in sealed serum bottles using sterile CO_2-flushed syringes and needles. A total of 0.1 mL of each dilution from the chosen range were inoculated into 60 Hungate tubes containing RM02 + GenRFV[25] and incubated at 39° C. Tubes that showed any turbidity were scored as positive for growth, subcultured into fresh RM02 media, examined microscopically for cell uniformity and morphology and stored at -85° C. This group of cultures included representatives of the unclassified R-7 and RC-9 gut groups as well as several bacteria that do not match currently described genera (Supplementary Table 1).

The initial culture survey highlighted the under-representation of members of the phylum Bacteroidetes[160], and targeted isolation experiments from samples of cow rumen contents were used to provide additional cultures specifically for this project. RM02 medium[25] with cellobiose, cellulose, galacturonic acid or pectin as the carbohydrate source, or RM02 medium supplemented with haemin and vitamin K (KH medium) were used to obtain 18 additional cultures from the families *Bacteroidaceae*, *Porphyromonadaceae* and *Prevotellaceae* within the Bacteroidetes phylum. These isolation experiments also provided new strains from the phyla Actinobacteria (13), Firmicutes (42), Proteobacteria (5) and Spirochaetes (1). These strains are available from the AgResearch culture collection and are marked as targeted isolates (TI) in Supplementary Table 1.

Overall the Hungate collection includes almost all the archaeal and bacterial cultures that have been described and named from the rumen. The only exceptions for which we were unable to obtain viable cultures or genomic DNA were rumen strains of *Howardella ureilytica*[162], *Syntrophococcus sucromutans*[163], *Micromonospora ruminantium*[164], *Clostridium sticklandii* (*Peptoclostridium*)[165], *Mannheimia ruminalis*[166] and *Oxalobacter formigenes*[167]. Since this project began a small number of novel rumen bacteria have been

Nature Biotechnology: doi:10.1038/nbt.4110

described including *Treponema ruminis*[168], *Megasphaera hexanoica*[169], *Actinomyces succiniciruminis* and *Actinomyces glycerinitolerans*[170].

While 86% (431) of the sequenced cultures were of established rumen origin a further 70 cultures originated from either feces (68) or saliva (the 2 *Alysiella* isolates from sheep saliva). It is known that fecal isolates are not a good proxy for the rumen[171,172] but this subset of cultures was included to provide additional information on the microbiota of the ruminant gastrointestinal tract. It is apparent that much of the human metagenome recruitment relates to this subset of fecal cultures.

The origin of the fecal isolates was as follows:

1. 16 were from newborn calf feces from before the rumen has developed and the animals are predominantly fed milk or a calf starter milk replacement. These cultures are probably a special case not truly representative of the rumen or the GI tract of an adult animal.
2. 38 cultures came from a USDA study (NLAE-zl strains) that isolated bacteria from fecal enrichments from cows (21 isolates), and goats (17 isolates)[10] . This is the only study that has attempted to isolate bacteria from ruminant feces in a systematic way.
3. 7 isolates were from cow feces. These include the type strains of *Methanobrevibacter thaueri* (known to be present in the rumen from 16S rRNA gene studies but no rumen isolates available), *Clostridium intestinale* and *Streptococcus equinus*.
4. 2 isolates were from horse feces included because they are representative of known rumen organisms for which no rumen isolates were available (*Methanobrevibacter gottschalkii* and *Sharpea azabuensis,* although we were later able to isolate rumen strains of the latter).
5. 1 isolate from lamb feces (*Peptoclostridium mangenotii*).
6. 1 isolate from deer feces (*Peptostreptococcaceae* bacterium with 99% match to *Romboutsia sedimentorum*).

Nature Biotechnology: doi:10.1038/nbt.4110

7. 1 isolate from pig feces-the type strain of *Methanobrevibacter gottschalkii* (known to be present in the rumen from 16S rRNA gene studies but no rumen isolates available).
8. 1 isolate from sheep feces-the type strain of *Methanobrevibacter wolinii* (known to be present in the rumen from 16S rRNA gene studies but no rumen isolates available-this culture is no longer available from DSM).
9. 1 isolate from goose feces-the type strain of *Methanobrevibacter woesii* (known to be present in the rumen from 16S rRNA gene studies but no rumen isolates available).

Cultures were grown according to referenced methods and media (see Supplementary Table 1 for references) or routinely resuscitated from frozen stocks in RM02 medium[25] and incubated at 39°C in the dark for up to 14 days or until turbid. All cultures were checked for purity by successive subculture from liquid culture to solid media. Individual colonies were purified by streaking onto fresh agar plates, and sub-cultured into liquid culture. If the cultures were true-to-label they were catalogued and stored at -85°C. Briefly, solid media were prepared by adding 7.5g agar to 500ml basal medium bubbled through with 100% CO_2 gas, and autoclaved. Agar plates were poured in an anaerobic glove box (93% CO_2, 7% H_2), and dried. Inoculated plates were placed in stainless steel canisters pressurized with CO_2 and incubated at 39°C for up to 14 days.

To identify novel cultures or confirm culture identity, DNA was extracted from 10ml RM02 cultures and subjected to partial 16S rRNA gene sequencing using the 27f and 1492r primers. Briefly bacterial cells were harvested, re-suspended in Tris EDTA (TE) buffer (pH 7.4). RNAse was added to give a final concentration of 140µg/ml. Bacteria were lysed using lysozyme (final concentration of 12.5mg/ml, 37°C for 45min), proteinase K (final concentration of 1mg/ml, 52°C for 60min) and SDS (final concentration 1% [w/v], 52°C for 45min. Cellular proteins were removed by mixing the clear cell suspension with phenol:chloroform:isopropanol (25:24:1) and removing the upper aqueous layer after

Nature Biotechnology: doi:10.1038/nbt.4110

centrifugation. This procedure was repeated and DNA precipitated with isopropanol, washed with 70% ethanol and resuspended in TE buffer.

SUPPLEMENTARY NOTE 2

Biosynthetic gene clusters

Biosynthetic gene clusters produce a diverse range of small molecules believed to mediate microbe-microbe and microbe-host interactions and some have been shown to have antimicrobial activity. To determine if rumen microbes produce a novel range of secondary metabolites the 501 genomes were screened for biosynthetic gene clusters using the antiSMASH 3.0 program[173]. A total of 6,906 biosynthetic clusters were predicted from the Hungate genomes (Supplementary Table 7). Results for bacteriocins, lantipeptides and non-ribosomal peptides (secondary metabolites known to have antimicrobial activity) are summarized in Supplementary Fig. 5. Of the 288 non-ribosomal peptide biosynthetic clusters detected, 251 were encoded by 153 Firmicute genomes belonging to 23 genera. Six out of 21 Euryarchaeal genomes also encode 10 non-ribosomal peptide biosynthetic clusters, and these are believed to have been acquired by horizontal gene transfer[148]. Bacteriocins from rumen bacteria have been described previously[174,175], and their use as rumen modifiers to reduce methane production in ruminants has been suggested[176]. It is not known if secondary metabolites from rumen bacteria have activity against rumen methanogens, but the bacteriocin nisin[177], and synthetic antimicrobial peptides[178] have been reported to be inhibitory to methanogens.

SUPPLEMENTARY NOTE 3

CRISPR-CAS system spacer analysis

The Hungate CRISPR spacer collection (6,344 spacer sequences predicted from 241 genomes) was searched against three databases: (i) IMG/VR[179], (ii) global “spacerome” comprised of spacers predicted from all isolates and metagenomes in the IMG database, and

Nature Biotechnology: doi:10.1038/nbt.4110

(iii) NBCI refseq plasmid database. Only 169 (2.7% of total) Hungate spacers had matches against IMG/VR, yielding novel linkages or association between 31 Hungate genomes and 83 non-redundant viruses (Supplementary Table 8). Expectedly, the majority (94%) of these viruses were derived from mammalian host-associated samples. More specifically, ~61% were from human intestinal (fecal) samples, 26% from rumen, 7.6% from human oral, and 5.7% from aquatic samples. The larger relative proportion of human intestinal viruses was primarily due to the preponderance of these types of samples relative to rumen samples, but the associated Hungate hosts were themselves of primarily ruminant fecal origin whereas rumen isolates were associated primarily with viruses predicted from rumen metagenomic samples.

A total of 821 (~13% of total) Hungate spacers possessed hits in the spacerome collection from isolate genomes (Supplementary Table 8). We observed that 85% of all the spacer matches were found between different strains of the same species and 4.6% from spacer matches between different species of the same genus, reflecting a high host-specificity for these mobile genetic elements. The remaining 10.4% of the hits were found between spacers from members of *Escherichia* spp. and *Shigella* spp. A single instance of identical spacer sequence between two members of different orders (*Enterobacteriales* and *Pseudomonadales*) was detected. About 380 Hungate spacers were detected in the spacerome arising from metagenomes, and exploring the habitat metadata for these revealed similar patterns as discussed above in the IMG/VR results. The search against the NBCI refseq plasmid database resulted in no significant hits.

SUPPLEMENTARY NOTE 4

Metagenome recruitment

About 129 Hungate isolates that were not detected in rumen metagenome samples (based on the recruitment constraints described in methods), did recruit other types of environmental

Nature Biotechnology: doi:10.1038/nbt.4110

samples in the IMG database (Fig.4, Supplementary Table 9). For example, *Pseudochrobactrum* sp. AO18b, *Bacillus cereus* KPR-7A, *Bacillus nealsonii* AAU1_buffalo and *Bordetella trematum* HR18 recruited several different plant rhizosphere samples, and might represent plant-associated bacteria ingested by grazing animals. These could be expected to be transient members of the microbiota rather than true denizens of the rumen. *Pediococcus acidilactici* AGR20 was detected exclusively in an insect (*Panchlora* sp.) gut sample, but pediococci are also commonly associated with forage crops and used as silage inoculants in the production of animal feeds[180]. *Enterobacter* sp. KPR-6, *Escherichia coli* PA-3, *Citrobacter* sp. NLAE-zl-C269, *Shigella sonnei* NLAE-zl-G496 were almost ubiquitously detected in hundreds of samples from aquatic, terrestrial, plant, insect, human and many other sources. *Basfia succiniciproducens* strains, *Streptococcus henryi* A-4, *Actinobacillus succinogenes* 130Z, and *Actinomyces ruminicola* strains were exclusively recruited by numerous human oral samples, *Bifidobacterium longum* AGR2137, *B. breve* RP2, and *B. bifidum* Calf96 were detected in human intestinal samples, and *Staphylococcus epidermidis* NLAE-zl-G239 recruited primarily nasopharyngeal and skin samples from the retroauricular crease. In all, 66 Hungate isolates were recruited by human samples (exclusively (20) or otherwise) and not detected in rumen samples.

As further validation of the protein sequence-based recruitment results, we also performed a nucleotide-based recruitment of >15 billion unassembled reads from selected rumen (sheep (20) and bovine (3)) and human stool (4) metagenome samples against the Hungate isolates (Supplementary Table 9). Overall, these results reaffirmed protein recruitment results as stated in the main text, with no perceived contradictions or additional evidence of recruitment; indeed, the same isolates were recognized as top recruiters using both approaches.

Nature Biotechnology: doi:10.1038/nbt.4110

SUPPLEMENTARY REFERENCES (for Supplementary Table 1, Supplementary Table 3 and Supplementary Text).

1. An, D., Cai, S., & Dong, X. *Actinomyces ruminicola* sp. nov., isolated from cattle rumen. *Int. J. Syst. Evol. Microbiol.* **56,** 2043-2048 (2006).
2. Kraatz, M., Wallace, R. J., & Svensson, L. *Olsenella umbonata* sp. nov., a microaerotolerant anaerobic lactic acid bacterium from the sheep rumen and pig jejunum, and emended descriptions of *Olsenella*, *Olsenella uli* and *Olsenella profusa*. *Int. J. Syst. Evol. Microbiol.* **61,** 795-803 (2011).
3. Scardovi, V., Trovatelli, L. D., Crociani, F., & Sgorbati, B. Bifid bacteria in bovine rumen. New species of the genus *Bifidobacterium*: *B. globosum* n. sp. and *B. ruminale* n.sp. *Arch. Mikrobiol*. **68,** 278-294 (1969).
4. Scardovi, V., Trovatelli, L. D., Biavati, B., & Zani, G. *Bifidobacterium cuniculi*, *Bifidobacterium choerinum*, *Bifidobacterium boum*, and *Bifidobacterium pseudocatenulatum*: four new species and their deoxyribonucleic acid homology relationships. *Int. J. Syst. Bacteriol.* **29,** 291-311 (1979).
5. Kelly, W. J. *et al.* Genomic analysis of three *Bifidobacterium* species isolated from the calf gastrointestinal tract. *Sci. Rep.* **6,** 30768 (2016).
6. Biavati, B., & Mattarelli, P. *Bifidobacterium ruminantium* sp. nov. and *Bifidobacterium merycicum* sp. nov. from the rumens of cattle. *Int. J. Syst. Bacteriol*. **41,** 163-168 (1991).
7. Anderson, R. C., Rasmussen, M. A., Jensen, N. S., & Allison, M. J. *Denitrobacterium detoxificans* gen. nov., sp. nov., a ruminal bacterium that respires on nitrocompounds. *Int. J. Syst. Evol. Microbiol*. **50,** 633-638 (2000).
8. Noel, S. *Cultivation and community composition analysis of plant-adherent rumen bacteria.* PhD thesis, Massey University, Palmerston North, New Zealand (2013).

Nature Biotechnology: doi:10.1038/nbt.4110

9. Klieve, A. V., Hudman, J. F. & Bauchop, T. Inducible bacteriophages from ruminal bacteria. *Appl. Environ. Microbiol.* **55,** 1630-1634 (1989).
10. Ziemer, C. J. Newly cultured bacteria with broad diversity isolated from 8 week continuous culture enrichments of cow feces on complex polysaccharides. *Appl. Environ. Microbiol.* **80,** 574-585 (2014).
11. Reilly, K., Carruthers, V. R., & Attwood, G. T. Design and use of 16S ribosomal DNA-directed primers in competitive PCRs to enumerate proteolytic bacteria in the rumen. *Microb. Ecol.* **43,** 259-270 (2002).
12. Nyonyo, T., Shinkai, T., Tajima, A., & Mitsumori, M. Effect of media composition, including gelling agents, on isolation of previously uncultured rumen bacteria. *Lett. Appl. Microbiol.* **56,** 63-70 (2013).
13. Dehority, B. A. Pectin-fermenting bacteria isolated from the bovine rumen. *J. Bacteriol.* **99,** 189-196 (1969).
14. Avguštin, G., Wright, F., & Flint, H.J. Genetic diversity and phylogenetic relationships among strains of *Prevotella* (*Bacteroides*) *ruminicola* from the rumen. *Int. J. Syst. Bacteriol.* **44,** 246-255 (1994).
15. Montgomery, L. *et al.* Transfer of *Bacteroides succinogenes* (Hungate) to *Fibrobacter* gen. nov. as *Fibrobacter succinogenes* comb. nov. and description of *Fibrobacter intestinalis* sp. nov. *Int. J. Syst. Bacteriol*. **38,** 430-435 (1988).
16. Wallace, R. J. *et al*. *Eubacterium pyruvativorans* sp. nov., a novel non-saccharolytic anaerobe from the rumen that ferments pyruvate and amino acids, forms caproate and utilizes acetate and propionate. *Int. J. Syst. Evol. Microbiol*. **53,** 965-970 (2003).
17. Van Gylswyk, N.O. *Succiniclasticum ruminis* gen. nov., sp. nov., a ruminal bacterium converting succinate to propionate as the sole energy-yielding mechanism. *Int. J. Syst. Bacteriol.* **45,** 297-300 (1995).

Nature Biotechnology: doi:10.1038/nbt.4110

18. Ishaq, S. L., Kim, C. J., Reis, D., & Wright, A. D. G. Fibrolytic bacteria Isolated from the rumen of North American moose (*Alces alces*) and their use as a probiotic in neonatal lambs. *PLoS One* **10,** e0144804 (2015).

19. Varel, V. H. Reisolation and characterization of *Clostridium longisporum,* a ruminal sporeforming cellulolytic anaerobe. *Arch. Microbiol.* **152,** 209-214 (1989).

20. Kelly, W. J., Asmundson, & R. V., Hopcroft, D. H. Isolation and characterization of a strictly anaerobic, cellulolytic spore former: *Clostridium chartatabidum* sp. nov. *Arch. Microbiol.* **147,** 169-173 (1987).

21. Cirne, D. G., Delgado, O. D., Marichamy, S., & Mattiasson, B. *Clostridium lundense* sp. nov., a novel anaerobic lipolytic bacterium isolated from bovine rumen. *Int. J. Syst. Evol. Microbiol.* **56,** 625-628 (2006).

22. Zhang, K., Song, L., & Dong, X. *Proteiniclasticum ruminis* gen. nov., sp. nov., a strictly anaerobic proteolytic bacterium isolated from yak rumen. *Int. J. Syst. Evol. Microbiol*. **60,** 2221-2225 (2010).

23. Hudson, J. A., MacKenzie, C. A. M., & Joblin, K. N. Conversion of oleic acid to 10-hydroxystearic acid by two species of ruminal bacteria. *Appl. Microbiol. Biotechnol.* **44,** 1-6 (1995).

24. Morvan, B., & Joblin, K. N. Hydration of oleic acid by *Enterococcus gallinarum, Pediococcus acidilactici* and *Lactobacillus* sp. *Anaerobe* **5,** 605-611 (2000).

25. Kenters, N., Henderson, G., Jeyanathan, J., Kittelmann, S., & Janssen, P.H. Isolation of previously uncultured rumen bacteria by dilution to extinction using a new liquid culture medium. *J. Microbiol. Methods* **84,** 52-60 (2011).

26. Bryant, M. P., Small, S. N., Bouma, C., & Robinson, I. Studies on the composition of the ruminal flora and fauna of young calves. *J. Dairy Sci*. **41,** 1747-1767 (1958).

Nature Biotechnology: doi:10.1038/nbt.4110

27. Sharpe, M. E., Latham, M. J., Garvie, E. I., Zirngibl, J., & Kandler, O. Two new species of *Lactobacillus* isolated from the bovine rumen, *Lactobacillus ruminis* sp. nov. and *Lactobacillus vitulinus* sp. nov. *J. Gen. Microbiol.* **77,** 37-49 (1973).

28. Salvetti, E. *et al.* Reclassification of *Lactobacillus catenaformis* (Eggerth 1935) Moore and Holdeman 1970 and *Lactobacillus vitulinus* Sharpe et al. 1973 as *Eggerthia catenaformis* gen. nov., comb. nov. and *Kandleria vitulina* gen. nov., comb. nov., respectively. *Int. J. Syst. Evol. Microbiol.* **61,** 2520-2524 (2011).

29. Attwood, G. T., Klieve, A. V., Ouwerkerk, D., & Patel, B. K. C. Ammonia-hyperproducing bacteria from New Zealand ruminants. *Appl. Environ. Microbiol.* **64,** 1796-1804 (1998).

30. Morita, H. *et al. Sharpea azabuensis* gen. nov., sp. nov., a Gram-positive, strictly anaerobic bacterium isolated from the faeces of thoroughbred horses. *Int. J. Syst. Evol. Microbiol.* **58,** 2682-2686 (2008).

31. Greening, R. C., & Leedle, J. A. Z. Enrichment and isolation of *Acetitomaculum ruminis*, gen. nov.: acetogenic bacteria from the bovine rumen. *Arch. Microbiol*. **151,** 399-406 (1989).

32. Rieu-Lesme, F., Morvan, B., Collins, M. D., Fonty, G., & Willems, A. A new H_2/CO_2-using acetogenic bacterium from the rumen: description of *Ruminococcus schinkii* sp. nov. *FEMS Microbiol. Lett*. **140,** 281-286 (1996).

33. Liu, C., Finegold, S. M., Song, Y., & Lawson, P. A. Reclassification of *Clostridium coccoides*, *Ruminococcus hansenii*, *Ruminococcus hydrogenotrophicus*, *Ruminococcus luti*, *Ruminococcus productus* and *Ruminococcus schinkii* as *Blautia coccoides* gen. nov., comb. nov., *Blautia hansenii* comb. nov., *Blautia hydrogenotrophica* comb. nov., *Blautia luti* comb. nov., *Blautia producta* comb. nov., *Blautia schinkii* comb. nov. and description

Nature Biotechnology: doi:10.1038/nbt.4110

of *Blautia wexlerae* sp. nov., isolated from human faeces. *Int. J. Syst. Evol. Microbiol*. **58,** 1896-1902 (2008).

34. Clarke, R. T. J., Bailey, R. W., & Gaillard, B. D. E. Growth of rumen bacteria on plant cell wall polysaccharides. *J. Gen. Microbiol.* **56,** 79-86. (1969).
35. Wallace, R. J. *et al*. *Clostridium proteoclasticum*: a ruminal bacterium that forms stearic acid from linoleic acid. *FEMS Microbiol. Lett.* **265,** 195-201 (2006).
36. Van de Vossenberg, J. L. C. M., & Joblin, K. N. Biohydrogenation of C18 unsaturated fatty acids to stearic acid by a strain of *Butyrivibrio hungatei* from the bovine rumen. *Lett. Appl. Microbiol.* **37,** 424-428 (2003).
37. Kalmoloff, M. L., Bartlett, F., & Teather, R. M. Are ruminal bacteria armed with bacteriocins? *J. Dairy Sci*. **79,** 2297-2306 (1996).
38. Ghali, M. B., Scott, P. T., Alhadrami, G. A., & Al Jassim, R. A. M. Identification and characterisation of the predominant lactic acid-producing and lactic acid-utilising bacteria in the foregut of the feral camel (*Camelus dromedarius*) in Australia. *Anim. Prod. Sci.* **51,** 597-604 (2011).
39. Krumholz, L. R. & Bryant, M. P. *Eubacterium oxidoreducens* sp. nov. requiring H_2 or formate to degrade gallate, pyrogallol, phloroglucinol and quercetin. *Arch. Microbiol*. **144,** 8-14 (1986).
40. Whitford, M. F., Yanke, L. J., Forster, R. J., & Teather, R. M. *Lachnobacterium bovis* gen. nov., sp. nov., a novel bacterium isolated from the rumen and faeces of cattle. *Int. J. Syst. Evol. Microbiol.* **51,** 1977-1981 (2001).
41. Van Gylswyk, N. O., & Van Der Toorn, J. J. T. K. *Clostridium aerotolerans* sp. nov., a xylanolytic bacterium from corn stover and from the rumina of sheep fed corn stover. *Int. J. Syst. Bacteriol.* **37,** 102-105 (1987).

Nature Biotechnology: doi:10.1038/nbt.4110

42. Paster, B. J. *et al*. Phylogeny of the ammonia-producing ruminal bacteria *Peptostreptococcus anaerobius, Clostridium sticklandii,* and *Clostridium aminophilum* sp. nov. *Int. J. Syst. Bacteriol.* 4**3,** 107-110 (1993).

43. Kaneuchi, C., Watanabe, K., Terada, A., Benno, Y., & Mitsuoka, T. Taxonomic study of *Bacteroides clostridiiformis* subsp. *clostridiiformis* (Burri and Ankersmit) Holdeman and Moore and of related organisms: Proposal of *Clostridium clostridiiformis* (Burri and Ankersmit) comb. nov. and *Clostridium symbiosum* (Stevens) comb. nov. *Int. J. Syst. Bacteriol*. **26,** 195-204 (1976).

44. Van Gylswyk, N. O., Morris, E. J., & Els, H. J. Sporulation and cell wall structure of *Clostridium polysaccharolyticum* comb. nov. (formerly *Fusobacterium polysaccharolyticum*). *J. Gen. Microbiol*. **121,** 491-493 (1980).

45. Bryant, M. P., & Small, N. Characteristics of two new genera of anaerobic curved rods isolated from the rumen of cattle. *J. Bacteriol*. **72,** 22-26 (1956).

46. Rosero, J. A. *et al*. Reclassification of *Eubacterium rectale* (Hauduroy et al. 1937) Prévot 1938 in a new genus *Agathobacter* gen. nov. as *Agathobacter rectalis* comb. nov., and description of *Agathobacter ruminis* sp. nov., isolated from the rumen contents of sheep and cows. *Int. J. Syst. Evol. Microbiol*. **66,** 768-773 (2016).

47. Lewis, S. M., & Dehority, B. A. Microbiology and ration digestibility in the hindgut of the ovine. *Appl. Environ. Microbiol.* **50,** 356-363 (1998).

48. Van Gylswyk, N. O., Hippe, H., & Rainey, F. A. *Pseudobutyrivibrio ruminis,* gen. nov., sp. nov., a butyrate-producing bacterium from the rumen that closely resembles *Butyrivibrio fibrisolvens* in phenotype. *Int. J. Syst. Bacteriol*. **46,** 559-563 (1996).

49. Klieve, A. V. *et al*. Establishing populations of *Megasphaera elsdenii* YE 34 and *Butyrivibrio fibrisolvens* YE 44 in the rumen of cattle fed high grain diets. *J. Appl. Microbiol*. **95,** 621-630 (2003).

Nature Biotechnology: doi:10.1038/nbt.4110

50. Sharpe, M. E., & Reiter, B. Common antigenic determinant in a rumen organism and in salmonellae containing the antigen 04. *Appl. Microbiol.* **24,** 613-617 (1972).

51. Mannarelli, B. M., Stack, R. J., Lee, D., & Ericsson, L. Taxonomic relatedness of *Butyrivibrio*, *Lachnospira*, *Roseburia* and *Eubacterium* species as determined by DNA hybridization and extracellular-polysaccharide analysis. *Int. J. Syst. Bacteriol.* **40,** 370-378 (1990).

52. Wallace, R. J., & Brammall, M. L. The role of different species of bacteria in the hydrolysis of protein in the rumen. *Microbiology* **131,** 821-832 (1985).

53. Bryant, M. P., & Small, N. The anaerobic monotrichous butyric acid-producing curved rod-shaped bacteria of the rumen. *J. Bacteriol.* **72,** 16-21 (1956).

54. Kopecny, J., Zorec, M., Mrázek, J., Kobayashi, Y., & Marinsek-Logar, R. *Butyrivibrio hungatei* sp. nov. and *Pseudobutyrivibrio xylanivorans* sp. nov., butyrate-producing bacteria from the rumen. *Int. J. Syst. Evol. Microbiol.* **53,** 201-209 (2003).

55. Van Gylswyk, N. O., & Van Doorn, C. E. A. Incidence and some growth characteristics of lactate-fermenting ruminal sarcinas. *Swedish J. Agric. Res.* **22,** 131-139 (1992).

56. Hudson, J. A., Cai, Y., Corner, R. J., Morvan, B., & Joblin, K. N. Identification and enumeration of oleic acid and linoleic acid hydrating bacteria in the rumen of sheep and cows. *J. Appl. Microbiol.* **88,** 286-292. (2000).

57. Collins, E. *Investigation of the rumen microbial community degrading the non-protein amino acid ADAB from Acacia angustissima.* PhD thesis, The University of Queensland, Australia (2009).

58. Koike, S. *et al.* Molecular monitoring and isolation of previously uncultured bacterial strains from the sheep rumen. *Appl. Environ. Microbiol.* **76,** 1887-1894 (2010).

Nature Biotechnology: doi:10.1038/nbt.4110

59. Klieve, A. V., O'Leary, M. N., McMillen, L., & Ouwerkerk, D. *Ruminococcus bromii*, identification and isolation as a dominant community member in the rumen of cattle fed a barley diet. *J. Appl. Microbiol*. **103,** 2065-73 (2007).

60. Sun, X. Z., Joblin, K. N. Andrew, I. G., Hoskin, S. O., & Harris, P. J. Degradation of forage chicory by ruminal fibrolytic bacteria. *J. Appl. Microbiol.* **105,** 1286-1297 (2008).

61. Krause, D. O., Dalrymple, B. P., Smith, W. J., Mackie, R. I., & McSweeney, C. S. 16s rDNA sequencing of *Ruminococcus albus* and *Ruminococcus flavefaciens*: design of a signature probe and its application in adult sheep. *Microbiology* **145,** 1797-1807 (1999).

62. Klieve, A. V. *et al*. Naturally occurring DNA transfer system associated with membrane vesicles in cellulolytic *Ruminococcus* spp. of ruminal origin. *Appl. Environ. Microbiol.* **71,** 4248-4253 (2005).

63. Kelly, W. J., Ward, L. J. H., & Leahy, S. C. Chromosomal diversity in *Lactococcus lactis* and the origin of dairy starter cultures. *Genome Biol. Evol.* **2,** 729-744 (2010).

64. Oxford, A. E. The nutritional requirements of rumen strains of *Streptococcus bovis* considered in relation to dextran synthesis from sucrose. *J. Gen. Microbiol*. **19,** 617-623 (1958).

65. Russell, J. B., & Baldwin, R. L. Substrate preferences in rumen bacteria: evidence of catabolite regulatory mechanisms. *Appl. Environ. Microbiol*. **36,** 319-329 (1978).

66. Marounck, M., & Wallace, R. J. Influence of culture Eh on the growth and metabolism of the rumen bacteria *Selenomonas ruminantium, Bacteroides amylophilus, Bacteroides succinogenes* and *Streptococcus bovis* in batch culture. *J. Gen. Microbiol*. **130,** 223-229 (1984).

67. Boyer, E. W. *Amylolytic enzymes and selected physiological properties of Streptococcus bovis and Streptococcus equinus*. PhD thesis, Iowa State University, USA (1969).

Nature Biotechnology: doi:10.1038/nbt.4110

68. Klieve, A. V., Heck, G. L., Prance, M. A., & Shu, Q. Genetic homogeneity and phage susceptibility of ruminal strains of *Streptococcus bovis* isolated in Australia. *Lett. Appl. Microbiol*. **29,** 108-112 (1999).

69. Brooker, J. D. *et al*. *Streptococcus caprinus* sp. nov., a tannin-resistent ruminal bacterium from feral goats. *Lett. Appl. Microbiol*. **18,** 313-318 (1994).

70. Sly, L. I., Cahill, M. M., Osawa, R., & Fujisawa, T. The tannin-degrading species *Streptococcus gallolyticus* and *Streptococcus caprinus* are subjective synonyms. *Int. J. Syst. Bacteriol*. **47,** 893-894 (1997).

71. Garner, M. R., Flint, J. F., & Russell, J. B. *Allisonella histaminiformans* gen. nov., sp. nov. *Syst. Appl. Microbiol.* **25,** 498-506 (2002).

72. Weimer, P. J., & Moen, G. N. Quantitative analysis of growth and volatile fatty acid production by the anaerobic ruminal bacterium *Megasphaera elsdenii* T81. *Appl. Microbiol. Biotechnol.* **97,** 4075-4081 (2013).

73. Ouwerkerk, D., Klieve, A. V., & Forster, R. J. Enumeration of *Megasphaera elsdenii* in rumen contents by real-time Taq nuclease assay. *J. Appl. Microbiol*. **92,** 753-758 (2002).

74. Lan, G. Q., Ho, Y. W., & Abdullah, N. *Mitsuokella jalaludinii* sp. nov., from the rumens of cattle in Malaysia. *Int. J. Syst. Evol. Microbiol*. **52,** 713-718 (2002).

75. Sawanon, S., Koike, S., & Kobayashi, Y. Evidence for the possible involvement of *Selenomonas ruminantium* in rumen fiber digestion. *FEMS Microbiol. Lett.* **325,** 170-179 (2011).

76. Huhtanen, C. N., & Gall, L. S. Rumen organisms I. Curved rods and a related rod type. *J. Bacteriol.* **65,** 548-553 (1953).

77. Bryant, M. P. The characteristics of strains of *Selenomonas* isolated from bovine rumen contents. *J. Bacteriol*. **72,** 162-167 (1956).

Nature Biotechnology: doi:10.1038/nbt.4110

78. Kuhnert, P., Scholten, E., Haefner, S. Mayor, D., & Frey, J. *Basfia succiniciproducens* gen. nov., sp. nov., a new member of the family *Pasteurellaceae* isolated from bovine rumen. *Int. J. Syst. Evol. Microbiol.* **60,** 44-50 (2010).

79. Stackebrandt, E., & Hippe, H. Transfer of *Bacteroides amylophilus* to a new genus *Ruminobacter* gen. nov., nom. rev., as *Ruminobacter amylophilus* comb. nov. *Syst. Appl. Microbiol*. **8,** 204-207 (1986).

80. Wilson, S. M. Some carbohydrate-fermenting organisms isolated from the rumen of the sheep. *J. Gen. Microbiol*. **9,** i-ii (1953).

81. Bryant, M. P. The isolation and characteristics of a spirochete from the bovine rumen. *J. Bacteriol*. **64,** 325-335 (1952).

82. Miller, T. L., Wolin, M. J., & Kusel, E. A. Isolation and characterization of methanogens from animal feces. *Syst. Appl. Microbiol*. **8,** 234-238 (1986).

83. Miller, T. L., & Lin, C. Description of *Methanobrevibacter gottschalkii* sp. nov., *Methanobrevibacter thaueri* sp. nov., *Methanobrevibacter woesei* sp. nov. and *Methanobrevibacter wolinii* sp. nov. *Int. J. Syst. Evol. Microbiol.* **52,** 819-822 (2002).

84. Rea, S. M., Bowman, J. P., Popovski, S., Pimm, C., & Wright, A.-D. G. *Methanobrevibacter millerae* sp. nov. and *Methanobrevibacter olleyae* sp. nov., methanogens from the ovine and bovine rumen that can utilize formate for growth. *Int. J. Syst. Evol. Microbiol*. **57,** 450-456 (2007).

85. Paynter, M. J. B., & Hungate, R. E. Characterization of *Methanobacterium mobile,* sp. n., isolated from the bovine rumen. *J. Bacteriol*. **95,** 1943-1951 (1968).

86. Al-Dilaimi, A., Albersmeier, A., Kalinowski, J., & Rückert, C. Complete genome sequence of *Corynebacterium vitaeruminis* DSM 20294T, isolated from the cow rumen as a vitamin B producer. *J. Biotechnol*. **189,** 70-71 (2014).

Nature Biotechnology: doi:10.1038/nbt.4110

87. Pukall, R. *et al*. Complete genome sequence of *Slackia heliotrinireducens* type strain (RHS 1). *Stand. Genomic Sci.* **1,** 234-241 (2009).

88. Kim, S. B., & Goodfellow, M. Reclassification of *Amycolatopsis rugosa* Lechevalier et al. 1986 as *Prauserella rugosa* gen. nov., comb. nov. *Int. J. Syst. Bacteriol.* **49,** 507-512 (1999).

89. Atherly, T. Genotypic and phenotypic comparison of *Bacteroides ovatus*, *B. thetaiotaomicron*, and *B. xylanisolvens* isolates obtained from cow, goat, human, and pig feces. MSc thesis, Iowa State University, USA (2014).

90. Shah, H. N., Collins, M. D., Olsen, I., Paster, B. J., & Dewhirst, F. E. Reclassification of *Bacteroides levii* (Holdeman, Cato, and Moore) in the genus *Porphyromonas*, as *Porphyromonas levii* comb. nov. *Int. J. Syst. Bacteriol*. **45,** 586-588 (1995).

91. Avguštin, G., Wallace, R.J., & Flint, H.J. Phenotypic diversity among ruminal isolates of *Prevotella ruminicola*: proposal of *Prevotella brevis* sp. nov., *Prevotella bryantii* sp. nov., and *Prevotella albensis* sp. nov. and redefinition of *Prevotella ruminicola*. *Int. J. Syst. Bacteriol.* **47,** 284-288 (1997).

92. Purushe, J. *et al.* Comparative genome analysis of *Prevotella ruminicola* and *Prevotella bryantii*: insights into their environmental niche. *Microb. Ecol*. **60,** 721-729 (2010).

93. Suen, G. *et al*. The complete genome sequence of *Fibrobacter succinogenes* S85 reveals a cellulolytic and metabolic specialist. *PLoS One*. **6,** e18814 (2011).

94. Nathani, N. M., Duggirala, S. M., Bhatt, V. D., Kapatel, J., & Joshi, C. G. Genomic analysis of a novel strain of *Bacillus nealsonii*, isolated from Surti buffalo rumen. *Adv. Biosci.Biotechnol*. **5,** 235-245 (2014).

95. Lee, W. K., Fujisawa, T., Kawamura, S., Itoh, K., & Mitsuoka, T. *Clostridium intestinalis* sp. nov., an aerotolerant species isolated from the feces of cattle and pigs. *Int. J. Syst. Bacteriol.* **39,** 334-336 (1989).

Nature Biotechnology: doi:10.1038/nbt.4110

96. Bengelsdorf, F. R., Poehlein, A., Schiel-Bengelsdorf, B., Daniel, R., & Dürre, P. Genome sequence of the acetogenic bacterium *Oxobacter pfennigii* DSM 3222T. *Genome Announc*. **3,** e01408-15 (2015).

97. Kelly, W. J. *et al*. The complete genome sequence of *Eubacterium limosum* SA11, a metabolically versatile rumen acetogen. *Stand Genomic Sci*. **11,** 26 (2016).

98. Palevich, N. Comparative genomics of *Butyrivibrio* and *Pseudobutyrivibrio* from the rumen. PhD thesis, Massey University, Palmerston North, New Zealand (2016).

99. Kelly, W.J. *et al*. The glycobiome of the rumen bacterium *Butyrivibrio proteoclasticus* B316(T) highlights adaptation to a polysaccharide-rich environment. *PLoS One*. **5,** e11942 (2010).

100. Cai, S., & Dong, X. *Cellulosilyticum ruminicola* gen. nov., sp. nov., isolated from the rumen of yak, and reclassification of *Clostridium lentocellum* as *Cellulosilyticum lentocellum* comb. nov. *Int. J. Syst. Evol. Microbiol*. **60,** 845-849 (2010).

101. Cai, S. *et al*. *Cellulosilyticum ruminicola*, a newly described rumen bacterium that possesses redundant fibrolytic-protein-encoding genes and degrades lignocellulose with multiple carbohydrate- borne fibrolytic enzymes. *Appl. Environ. Microbiol*. **76,** 3818-3824 (2010).

102. Van Gylswyk, N. O., & Van der Toorn, J. J. T. K. Description and designation of a neotype strain of *Eubacterium cellulosolvens* (*Cillobacterium cellulosolvens* Bryant, Small, Bouma and Robinson) Holdeman and Moore. *Int. J. Syst. Bacteriol.* **36,** 275-277 (1986).

103. Bryant, M. P. Bacterial species of the rumen. *Bacteriol. Rev.* **23,** 125-153 (1959).

104. Van Gylswyk, N. O., & Van Der Toorn, J. J. T. K. *Eubacterium uniforme* sp. nov. and *Eubacterium xylanophilum* sp. nov., fiber-digesting bacteria from the rumina of sheep fed corn stover. *Int. J. Syst. Bacteriol*. **35,** 323-326 (1985).

Nature Biotechnology: doi:10.1038/nbt.4110

105. Palop, M. L., Valles, S., Pinaga, F., & Flors, A. Isolation and characterization of anaerobic, cellulolytic bacterium, *Clostridium celerecrescens* sp. nov. *Int. J. Syst. Bacteriol*. **39,** 68-71 (1989).

106. Forde, B. M. *et al*. Genome sequences and comparative genomics of two *Lactobacillus ruminis* strains from the bovine and human intestinal tracts. *Microb. Cell Fact.* **10(Suppl 1),** S13 (2011).

107. Lee, G. H. *et al*. Genome sequence of *Oscillibacter ruminantium* strain GH1, isolated from rumen of Korean native cattle. *J. Bacteriol*. **194,** 6362 (2012).

108. Coleman, G. S. A sulphate-reducing bacterium from the sheep rumen. *J. Gen. Microbiol.* **22,** 423-436 (1960).

109. Spring, S. *et al*. Complete genome sequence of the sulfate-reducing firmicute *Desulfotomaculum ruminis* type strain (DL(T)). *Stand. Genomic Sci*. **7,** 304-319 (2012).

110. Nathani, N. M., Duggirala, S. M., M, C. S., Kothari, R. K., & Joshi, C. G. Isolation of chitinolytic *Clostridium* sp. NCR from Mehsani buffalo rumen, its genomic analysis and potential role in rumen. *Genom. Data*. **5,** 109-111 (2015).

111. Hungate, R. E. Studies on cellulose fermentation: I. The culture and physiology of an anaerobic cellulose-digesting bacterium. *J. Bacteriol*. **48,** 499-513 (1944).

112. Bryant, M. P., Small, N., Bouma, C., & Robinson, I. M. Characteristics of ruminal anaerobic cellulolytic cocci and *Cillobacterium cellulosolvens* n. sp. *J. Bacteriol*. **76,** 529-537 (1958).

113. Suen, G. *et al*. Complete genome of the cellulolytic ruminal bacterium *Ruminococcus albus* 7. *J. Bacteriol*. **193,** 5574-5575 (2011).

114. Hungate, R. E., & Stack, R. J. Phenylpropanoic acid: growth factor for *Ruminococcus albus*. *Appl. Environ. Microbiol*. **44,** 79-83 (1982).

Nature Biotechnology: doi:10.1038/nbt.4110

115. Dassa, B. *et al*. Rumen cellulosomics: divergent fiber-degrading strategies revealed by comparative genome-wide analysis of six ruminococcal strains. *PLoS One*. **9,** e99221 (2014).

116. Berg Miller, M. E. *et al*. Diversity and strain specificity of plant cell wall degrading enzymes revealed by the draft genome of *Ruminococcus flavefaciens* FD-1. *PLoS One*. **4,** e6650 (2009).

117. Berg Miller, M. E. *et al*. Phage-bacteria relationships and CRISPR elements revealed by a metagenomic survey of the rumen microbiome. *Environ. Microbiol.* **14,** 207-227 (2012).

118. Cavanagh, D. *et al*. Evaluation of *Lactococcus lactis* isolates from nondairy sources with potential dairy applications reveals extensive phenotype-genotype disparity and implications for a revised species. *Appl. Environ. Microbiol*. **81,** 3961-3972 (2015).

119. Benahmed, F. H. *et al*. Draft genome sequences of *Streptococcus bovis* strains ATCC 33317 and JB1. *Genome Announc*. **2,** e01012-14 (2014).

120. Azevedo, A. C., Bento, C. B., Ruiz, J. C., Queiroz, M. V., & Mantovani, H. C. Draft genome sequence of *Streptococcus equinus* (*Streptococcus bovis*) HC5, a lantibiotic producer from the bovine rumen. *Genome Announc*. **3,** e00085-15 (2015).

121. Privé, F. *et al*. Identification and characterization of three novel lipases belonging to families II and V from *Anaerovibrio lipolyticus* 5ST. *PLoS One*. **8,** e69076 (2013).

122. Marx, H. *et al*. Genome sequence of the ruminal bacterium *Megasphaera elsdenii*. *J. Bacteriol.* **193,** 5578-5579 (2011).

123. Van Gylswyk, N. O., Hippe, H., & Rainey, F.A. *Schwartzia succinivorans* gen. nov., sp. nov., another ruminal bacterium utilizing succinate as the sole energy source. *Int. J. Syst. Bacteriol.* **47**, 155-159 (1997).

Nature Biotechnology: doi:10.1038/nbt.4110

124. Zhang, K., & Dong, X. *Selenomonas bovis* sp. nov., isolated from yak rumen contents. *Int. J. Syst. Evol. Microbiol.* **59,** 2080-2083 (2009).

125. Kaneko, J. *et al*. Complete genome sequence of *Selenomonas ruminantium* subsp. *lactilytica* will accelerate further understanding of the nature of the class *Negativicutes*. *FEMS Microbiol. Lett.* **362,** fnv050 (2015).

126. Chang, D-H. *et al*. Draft genome sequence of *Bordetella trematum* strain HR18. *Genome Announc*. **3,** e01357-14 (2015).

127. Merga, J. Y., Winstanley, C., Williams, N. J., Yee, E., & Miller, W. G. Complete genome sequence of the *Arcobacter butzleri* cattle isolate 7h1h. *Genome Announc*. **1,** e00655-13 (2013).

128. Lee, N. *et al.* Emended description of the species *Lampropedia hyalina*. *Int. J. Syst. Evol. Microbiol.* **54,** 1709-1715 (2004).

129. Forsberg, C. W. Sulfide production from cysteine by *Desulfovibrio desulfuricans*. *Appl. Environ. Microbiol*. **39,** 453-455 (1980).

130. Baar, C. *et al*. Complete genome sequence and analysis of *Wolinella succinogenes*. *Proc. Natl. Acad. Sci. USA*. **100,** 11690-11695 (2003).

131. Chang, D. H., Rhee, M. S., Jeong, H., Kim, S., & Kim, B. C. Draft genome sequence of *Acinetobacter* sp. HR7, isolated from Hanwoo, Korean native cattle. *Genome Announc*. **3,** e01358-14 (2015).

132. Xie, C. H., & Yokota, A. Phylogenetic analysis of *Alysiella* and related genera of *Neisseriaceae*: proposal of *Alysiella crassa* comb. nov., *Conchiformibium steedae* gen. nov., comb. nov., *Conchiformibium kuhniae* sp. nov. and *Bergeriella denitrificans* gen. nov., comb. nov. *J. Gen. Appl. Microbiol*. **51,** 1-10 (2005).

133. McKinlay, J. B. *et al*. A genomic perspective on the potential of *Actinobacillus succinogenes* for industrial succinate production. *BMC Genomics*. **11,** 680 (2010).

Nature Biotechnology: doi:10.1038/nbt.4110

134. Hong, S. H. *et al.* The genome sequence of the capnophilic rumen bacterium *Mannheimia succiniciproducens*. *Nat. Biotechnol.* **22,** 1275-1281 (2004).

135. Bryant, M. P., Small, N., Bouma, C., & Chu, H. *Bacteroides ruminicola* sp. nov. and *Succinomonas amylolytica* gen. nov. species of succinic acid-producing anaerobic bacteria of the bovine rumen. *J. Bacteriol*. **76,** 15-23 (1958).

136. Holland-Moritz, H. E. *et al*. Draft genome sequence of the pyridinediol-fermenting bacterium *Synergistes jonesii* 78-1. *Genome Announc*. **2,** e00833-14 (2014).

137. Davis, C. K., Webb, R. I., Sly, L. I., Denman, S. E., & McSweeney, C. S. Isolation and survey of novel fluoroacetate-degrading bacteria belonging to the phylum Synergistetes. *FEMS Microbiol. Ecol.* **80,** 671-684 (2012).

138. Paster, B. J., & Canale-Parola, E. *Treponema saccharophilum* sp. nov., a large pectinolytic spirochete from the bovine rumen. *Appl. Environ. Microbiol*. **50,** 212-219 (1985).

139. Rosewarne, C. P. *et al.* Draft genome sequence of *Treponema* sp. strain JC4, a novel spirochete isolated from the bovine rumen. *J. Bacteriol.* **194,** 4130 (2012).

140. Kelly, W. J. *et al*. Complete genome sequence of methanogenic archaeon ISO4-G1, a member of the *Methanomassiliicoccales*, isolated from a sheep rumen. *Genome Announc.* **4,** e00221-16 (2016).

141. Li, Y. *et al.* The complete genome sequence of the methanogenic archaeon ISO4-H5 provides insights into the methylotrophic lifestyle of a ruminal representative of the *Methanomassiliicoccales*. *Stand. Genomic Sci.* **11,** 59 (2016).

142. Kelly, W. J. *et al.* The complete genome sequence of the rumen methanogen *Methanobacterium formicicum* BRM9. *Stand. Genomic Sci.* **9,** 15 (2014).

143. Leahy, S. C. *et al*. The complete genome sequence of *Methanobrevibacter* sp. AbM4. *Stand. Genomic Sci.* **8,** 215-227 (2013).

Nature Biotechnology: doi:10.1038/nbt.4110

144. Lee, J-H. *et al*. Genome sequence of *Methanobrevibacter* sp. strain JH1, isolated from rumen of Korean native cattle. *Genome Announc.* **1,** e00002-13 (2013).

145. Lee, J-H. *et al*. *Methanobrevibacter boviskoreani* sp. nov., isolated from the rumen of Korean native cattle. *Int. J. Syst. Evol. Microbiol*. **63,** 4196-4201 (2013).

146. Kelly, W. J. *et al*. The complete genome sequence of the rumen methanogen *Methanobrevibacter millerae* SM9. *Stand. Genomic Sci.* **11,** 49 (2016).

147. Kelly, W. J. *et al*. Draft genome sequence of the rumen methanogen *Methanobrevibacter olleyae* YLM1. *Genome Announc.* **4,** e00232-16 (2016).

148. Leahy, S. C. *et al*. The genome sequence of the rumen methanogen *Methanobrevibacter ruminantium* reveals new possibilities for controlling ruminant methane emissions. *PLoS One*. **5,** e8926 (2010).

149. Lambie, S. C. *et al*. The complete genome sequence of the rumen methanogen *Methanosarcina barkeri* CM1. *Stand. Genomic Sci*. **10,** 57 (2015).

150. Mukherjee, S. *et al*. 1,003 reference genomes of bacterial and archaeal isolates expand coverage of the tree of life. *Nat. Biotechnol*. **35,** 676-683 (2017).

151. Palakawong Na Ayudthaya, S., Strepis, N., Pristaš, P., & Plugge, C. M. Draft genome sequence of *Actinomyces glycerinitolerans* strain G10^T, isolated from sheep rumen fluid. *Genome Announc.* **5,** e01589-16 (2017).

152. Palakawong Na Ayudthaya, S., Hornung, B., Ravikumar Varadarajan, A., Plugge, W., & Plugge, C. M. Draft genome sequence of *Actinomyces succiniciruminis* strain Am4^T, isolated from cow rumen fluid. *Genome Announc*. **5,** e01587-16 (2017).

153. Youssef, N. H. *et al*. The genome of the anaerobic fungus *Orpinomyces* sp. strain C1A reveals the unique evolutionary history of a remarkable plant biomass degrader. *Appl. Environ. Microbiol*. **79,** 4620-4634 (2013).

Nature Biotechnology: doi:10.1038/nbt.4110

154. Haitjema, C. H. *et al.* A parts list for fungal cellulosomes revealed by comparative genomics. *Nat. Microbiol*. **2,** 17087 (2017).

155. Gilbert, R. A. *et al.* Towards understanding phage:host interactions in the rumen; complete genome sequences of lytic phages infecting rumen bacteria. *Front. Microbiol.* doi: 10.3389/fmicb.2017.02340 (2017).

156. Hess, M. *et al.* Metagenomic discovery of biomass-degrading genes and genomes from cow rumen. *Science*. **331,** 463-467 (2011).

157. Luciano, T. *et al*. Metagenomic assembly and draft genome sequence of an uncharacterized *Prevotella* sp. from Nelore rumen. *Genome Announc*. **3,** 00723-15 (2015).

158. Svartström, O. *et al*. Ninety-nine de novo assembled genomes from the moose (*Alces alces*) rumen microbiome provide new insights into microbial plant biomass degradation. *ISME J.* **11,** 2538-2551 (2017).

159. Stewart, R., *et al*. Assembly of hundreds of microbial genomes from the cow rumen reveals novel microbial species encoding enzymes with roles in carbohydrate metabolism. bioRxiv 162578 (2017).

160. Creevey, C. J., Kelly, W. J., Henderson, G., & Leahy, S. C. Determining the culturability of the rumen microbiome. *Microb. Biotechnol.* **7**, 467-479 (2014).

161. Noel, S. J. *et al*. Seasonal changes in the digesta-adherent rumen bacterial communities of dairy cattle grazing pasture. *PLoS One*. **12,** e0173819 (2017).

162. Cook, A.R. *et al. Howardella ureilytica* gen. *nov.*, sp. *nov.*, a Gram-positive, coccoid-shaped bacterium from a sheep rumen. *Int. J. Syst. Evol. Microbiol*. **57**, 2940-2945 (2007).

Nature Biotechnology: doi:10.1038/nbt.4110

163. Krumholz, I. R. & Bryant, M.P. *Syntrophococcus sucromutans* sp. nov. gen. nov. uses carbohydrates as electron donors and formate, methoxymonobenzenoids or *Methanobrevibacter* as electron acceptor systems. *Arch. Microbiol.* **143,** 313-318 (1986).

164. Maluszyńska, G.M. & Janota-Bassalik, L. A cellulolytic rumen bacterium, *Micromonospora ruminantium* sp.nov. *J. Gen. Microbiol.* **82**, 57-65 (1974).

165. Paster, B. J. *et al.* Phylogeny of the ammonia-producing ruminal bacteria *Peptostreptococcus anaerobius*, *Clostridium sticklandii*, and *Clostridium aminophilum* sp. nov. *Int J Syst Bacteriol.* **43**, 107-110 (1993).

166. Angen, O., Mutters, R., Caugant, D. A., Olsen, J. E., & Bisgaard, M. Taxonomic relationships of the [*Pasteurella*] *haemolytica* complex as evaluated by DNA-DNA hybridizations and 16S rRNA sequencing with proposal of *Mannheimia haemolytica* gen. nov., comb, nov., *Mannheimia granulomatis* comb. nov., *Mannheimia glucosida* sp. nov., *Mannheimia ruminalis* sp. nov. and *Mannheimia varigena* sp. nov.. *Int. J. Syst. Evol. Microbiol*. **49**, 67–86 (1999).

167. Allison, M.J., Dawson, K.A., Mayberry, W.R., & Foss JG. *Oxalobacter formigenes* gen. nov., sp. nov.: oxalate-degrading anaerobes that inhabit the gastrointestinal tract. *Arch Microbiol*. **141**, 1-7 (1985).

168. Newbrook, K. *et al. Treponema ruminis* sp. nov., a spirochaete isolated from the bovine rumen. *Int. J. Syst. Evol. Microbiol.* **67**, 1349-1354 (2017).

169. Jeon, B. S., Kim, S., & Sang, B. I. *Megasphaera hexanoica* sp. nov., a medium-chain carboxylic acid-producing bacterium isolated from a cow rumen. *Int. J. Syst. Evol. Microbiol.* doi: 10.1099/ijsem.0.001888 (2017).

170. Palakawong Na Ayudthaya, S. *et al. Actinomyces succiniciruminis* sp. nov. and *Actinomyces glycerinitolerans* sp. nov., two novel organic acid-producing bacteria isolated from rumen. *Syst Appl Microbiol.* **39,** 445-452 (2016).

Nature Biotechnology: doi:10.1038/nbt.4110

171. Kim, M., & Wells, J. E. A meta-analysis of bacterial diversity in the feces of cattle. Curr. Microbiol. 72, 145-151 (2016).

172. Al-Masaudi, S. *et al.* A metagenomics investigation of carbohydrate-active enzymes along the gastrointestinal tract of Saudi sheep. *Front. Microbiol.* **8,** 666 (2017).

173. Weber, T. et al. antiSMASH 3.0-a comprehensive resource for the genome mining of biosynthetic gene clusters. *Nucleic Acids Res.* **43**, W237-243 (2015).

174. Kalmoloff, M. L., Bartlett, F., & Teather, R. M. Are ruminal bacteria armed with bacteriocins? *J. Dairy Sci*. **79,** 2297-2306 (1996).

175. Azevedo, A. C., Bento, C. B., Ruiz, J. C., Queiroz, M. V., & Mantovani, H. C. Distribution and genetic diversity of bacteriocin gene clusters in rumen microbial genomes. *Appl. Environ. Microbiol.* **81,** 7290-7304 (2015).

176. Knapp, J. R., Laur, G. L., Vadas, P. A., Weiss, W. P., & Tricarico, J. M. Invited review: Enteric methane in dairy cattle production: quantifying the opportunities and impact of reducing emissions. *J. Dairy Sci.* **97,** 3231-3261 (2014).

177. Hammes, W. P., Winter, J., & Kandler, O. The sensitivity of the pseudomurein-containing genus Methanobacterium to inhibitors of murein synthesis. *Arch. Microbiol.* **123,** 275-279 (1979).

178. Bang, C. *et al.* Effects of antimicrobial peptides on methanogenic archaea. *Antimicrob. Agents Chemother.* **56,** 4123-4130 (2012).

179. Paez-Espino, D. *et al.* IMG/VR: a database of cultured and uncultured DNA Viruses and retroviruses. *Nucleic Acids Res.* **45,** D457-D465 (2017).

180. Cai, Y., Kumai, S., Ogawa, M., Benno, Y., & Nakase, T. Characterization and identification of *Pediococcus* species isolated from forage crops and their application for silage preparation. *Appl. Environ. Microbiol.* **65,** 2901–2906 (1999).

Nature Biotechnology: doi:10.1038/nbt.4110
